# The Vaginal Microbiome Across the Continuum from HPV Infection to Cervical Cancer: A Narrative Review

**DOI:** 10.3390/microorganisms14102158

**Published:** 2026-09-24

**Authors:** Xiaodie Zhou, Jiaqi Hu, Ruoyu Zhou, Yi Lu, Xuan Du, Yipei Chen, Qingling Ren, Yue Han

**Affiliations:** 1 First Clinical Medical College, Nanjing University of Chinese Medicine, Nanjing 210023, China; zhouxiaodie2024@163.com (X.Z.);; 2 Jiangsu Province Hospital of Chinese Medicine, Affiliated Hospital of Nanjing University of Chinese Medicine, Nanjing 210029, China

**Keywords:** vaginal dysbiosis, high-risk human papillomavirus, cervical intraepithelial neoplasia, cervical cancer, Lactobacillus, microecological intervention, mucosal immunity, microbiome biomarker

## Abstract

Persistent high-risk human papillomavirus (HR-HPV) infection serves as the core prerequisite for cervical intraepithelial neoplasia (CIN) and invasive cervical cancer. Accumulated translational evidence confirms that vaginal microecological homeostasis dominated by protective Lactobacillus strains fundamentally governs the initiation, persistence, and spontaneous clearance of HR-HPV infection, while microbial dysbiosis featuring depleted lactobacilli and overgrown anaerobic pathogens creates a pro-inflammatory, immune-suppressive mucosal niche that accelerates viral retention and cervical malignant transformation. We conducted a narrative review of studies published primarily between January 2021 and April 2026. PubMed, Web of Science, CNKI, Wanfang Data, and VIP were searched using terms related to the vaginal microbiome, HR-HPV infection, CIN, cervical cancer, underlying mechanisms, microbial biomarkers, and microbiome-modulating interventions. Relevant peer-reviewed epidemiological, mechanistic, and clinical studies were prioritized, and the manuscript was prepared with reference to the Scale for the Assessment of Narrative Review Articles (SANRA). This review systematically delineates the bidirectional regulatory crosstalk between vaginal microbiota and HR-HPV across the cervical cancer continuum. Dysbiosis disrupts vaginal acidic barriers, reshapes local cytokine networks, impairs antiviral innate immunity, and modulates HPV E6/E7 oncogenic transcription; reciprocally, HR-HPV oncoproteins remodel microbial community structures to sustain a dysbiotic feedback loop. Three core carcinogenic cascades induced by dysbiotic flora are elaborated: epithelial barrier erosion, metabolic reprogramming, and epigenetic rewiring of host and viral genomes. Dynamic microbial characteristics corresponding to all stages of progressive cervical lesions, from transient HPV infection to high-grade CIN and carcinoma, are sorted out. The bench-to-bedside translational progress of microbiome-targeted interventions is summarized, including single/multi-strain probiotic supplementation, engineered lactic acid bacterial therapeutic vaccines, vaginal microbiota transplantation, and traditional Chinese medicine therapy that reconstructs vaginal eubiosis. The clinical application of microbial diversity, strain-level single-nucleotide variants, and taxonomic biomarkers for early screening, efficacy prediction, and postoperative recurrence risk stratification is illustrated. This comprehensive framework with the vaginal microbiome as the core clarifies the internal mechanism of HR-HPV progression along the entire pathway to cervical cancer and provides practical clinical means to block this cascade. Current bottlenecks, including inconsistent probiotic standards, preclinical model limitations, and unresolved gut–vagina microbiome interactions, require further investigation before widespread clinical translation.

## 1. Introduction

In 1892, Donderlein first reported the Lactobacillus-dominant configuration in the vaginal microbiome [1]. At present, a large number of studies have confirmed that this structure has emerged as a critical determinant of women’s health. The composition of its flora is closely related to a variety of health problems, including common gynecological infections, fertility, pregnancy status, and even systemic diseases [2,3,4,5,6,7,8]. The relevance of microbiome alterations may extend beyond cervical carcinogenesis to other gynecological malignancies. Aquino et al. [9] reviewed differences in microbiota composition between patients with endometrial cancer and healthy individuals and highlighted potential interactions among microbial dysbiosis, inflammation, metabolic factors, and the gynecological tumor microenvironment. These data, however, remain heterogeneous and largely associative, and tumor-specific microbial signatures and causal pathways require further validation. Interventions targeting the vaginal microbiome are moving into clinical investigation for common vaginal disorders. A recent randomized trial, for example, reported that cranberry-derived type A proanthocyanidins, given in addition to standard antifungal therapy, improved outcomes in vulvovaginal candidiasis [10]. Whether similar modulation can be harnessed for high-risk HPV (HR-HPV) infection and cervical carcinogenesis has not, however, been comprehensively synthesized. This paper mainly focuses on HPV infection and cervical cancer. Based on the existing bench to bedside research evidence, it aims to propose an intervention program based on the vaginal microbiome for the clinical prevention and treatment of HPV infection and cervical cancer.

## 2. Methods

This narrative review was based on a structured search of PubMed, Web of Science Core Collection, China National Knowledge Infrastructure (CNKI), Wanfang Data, and VIP for studies published from 1 January 2021 to 30 April 2026. Search terms included combinations of “vaginal microbiome”, “vaginal microbiota”, “vaginal dysbiosis”, “high-risk human papillomavirus”, “cervical intraepithelial neoplasia”, and “cervical cancer”, together with terms related to immunity, inflammation, metabolomics, microbial biomarkers, probiotics, and microbiome-targeted interventions. Corresponding Chinese terms were used in Chinese databases. Peer-reviewed epidemiological, clinical, and mechanistic studies directly relevant to the vaginal microbiome–HR-HPV–cervical carcinogenesis axis were considered. Selection was based on relevance, methodological quality, and contribution to mechanistic or translational understanding. Publications in journals with an impact factor above 5 and journals ranked primarily in Journal Citation Reports Q1 or Q2 were prioritized. However, these journal-level metrics were not used as absolute exclusion criteria, and highly relevant, seminal, or reviewer-recommended studies were also included. Duplicate records, conference abstracts without sufficient data, non-peer-reviewed publications, and studies with limited relevance or inadequate methodological information were excluded.

Owing to heterogeneity in study populations, sampling procedures, sequencing platforms, and outcomes, the evidence was synthesized narratively rather than quantitatively. The EQUATOR Network was consulted for an applicable reporting framework. As this was a narrative review, PRISMA was not formally applied; instead, manuscript preparation and revision were guided by the Scale for the Assessment of Narrative Review Articles (SANRA). Reference management was performed using Zotero (Corporation for Digital Scholarship, Vienna, VA, USA). Figures were generated using BioRender (Science Suite Inc., Toronto, ON, Canada) and BioGDP (Sun Yat-sen University, Guangzhou, China).

## 3. Vaginal Microbiome and HPV Infection: From Bench to Bedside

Persistent infection with high-risk human papillomavirus (HR-HPV) is a key driver of cervical cancer and its associated precancerous lesions [11,12]. The vaginal microbiome is the core basis for maintaining vaginal microecological homeostasis, which can regulate the complete development of HR-HPV infection, including the whole process from persistent infection to final clearance. A healthy vaginal microbiome dominated by Lactobacillus can resist HR-HPV infection, and the disorder of flora and the increase in microbial diversity will increase the risk of persistent virus infection and lesion progression. A large number of studies have shown that specific vaginal flora can eliminate the HPV virus by regulating the local immune microenvironment [13,14].

### 3.1. Vaginal Microbiome Dysbiosis Promotes HPV Infection and Its Persistence

Imbalance of the vaginal microbiome, also known as CST type IV, is an important incentive for the human body to be more susceptible to HPV infection and develop persistent infection [15,16,17]. Lactobacilli colonized in the vaginal epithelium decomposes glycogen to produce lactic acid, maintaining the vaginal pH below 4.5. This acidic environment can directly inhibit the excessive proliferation of conditional pathogenic bacteria in the microecological niche [18,19]. Lactobacilli also secrete active substances such as H_2_O_2_, bacteriocins, and antimicrobial peptides [20,21,22], which destroy the structure of pathogenic bacteria and inhibit the proliferation of pathogenic bacteria.

Lactobacilli can also compete with potentially pathogenic bacteria for nutrients and epithelial adhesion sites. After the colonization of these microorganisms on the vaginal mucosa surface, they prevent pathogenic bacteria from adhering to the mucosa and the subsequent invasion of pathogenic bacteria into human tissues [23,24]. Lactobacilli and its metabolites will further act on epithelial cells and immune cells, on the one hand, to promote the body to synthesize more antimicrobial peptides, and on the other hand, to regulate the response level of local cytokines [25]. The cytokines involved in such interactions include IL-1β, IL-6, IL-8, IL-2, IL-10, and IL-17; however, their expression patterns and physiological functions will change with different Lactobacillus strains, microbial metabolite profiles, host physical conditions, and local immune environments [26].

On the whole, the above mechanisms can maintain mucosal immune homeostasis and protect the reproductive tract from long-term inflammatory damage. Once the vaginal microbial homeostasis is broken, there is usually a decrease in the number of Lactobacillus as well as a decrease in the content of lactic acid and other protective metabolites.

These changes will destroy the integrity of the cervical vaginal epithelial barrier and make the tissue more susceptible to microbial and viral invasion. The decrease in lactic acid secretion will increase the vaginal pH value, and the antibacterial protective effect brought by the acidic microenvironment will also be weakened [27,28]. The overgrowth of anaerobic bacteria further sustains mucosal inflammation by triggering the release of pro-inflammatory cytokines and chemokines. Multiple microbes enriched under dysbiotic conditions also secrete mucin-degrading enzymes, such as sialidases, mucinases, and glycosidases. These enzymes break down mucosal mucins and compromise the structural stability of the protective mucus layer. Such structural damage enables HPV to penetrate epithelial gaps and access basal epithelial cells more efficiently [29]. Vaginal dysbiosis is typically defined by the loss of dominant Lactobacillus species and the overgrowth of anaerobic bacterial populations, such as *Gardnerella*, *Prevotella*, and *Fannyhessea vaginae* (previously classified as *Atopobium vaginae*) [30]. Increased bacterial diversity and anaerobic bacterial abundance have been associated with the enhanced production of inflammatory cytokines and chemokines, leading to immune-cell recruitment and persistent mucosal inflammation [31,32,33,34]. Dysregulated activation of mucosal immune cells may alter the local concentrations of inflammatory and immunomodulatory mediators, including IL-6, IL-8, TNF-α, TGF-β, MIP-1α, GM-CSF, IL-1α, and IL-1β. In parallel, the reduced production of antiviral mediators such as IL-2 and IL-12 may impair local cell-mediated immunity [35,36]. The resulting immune imbalance will make natural killer cells and cytotoxic T lymphocytes unable to properly identify and remove HPV-infected cells, thereby creating an environment conducive to the long-term survival of the virus [37,38]. Vaginal dysbiosis also interferes with pattern-recognition receptor-mediated antiviral defense mechanisms. Specifically, the downregulation of TLR3 expression or the blocking of signal transduction will result in the insufficient secretion of type I interferons such as IFN-α and IFN-β. After the effect of this pathway is weakened, the body’s inherent antiviral ability decreases, and HPV is more likely to achieve immune escape [39]. Based on the above mechanisms, it can be seen that vaginal dysbiosis will simultaneously destroy the physical and chemical barrier, epithelial structural integrity, and local immune surveillance system, and jointly promote HPV persistent infection from multiple pathways (see Figure 1).

However, HPV infection not only passively accepts the regulation of the flora, but also in turn reshapes the vaginal microbiome: high-risk HPV can encode E6 and E7 oncoproteins, which interfere with the NF-κB and TLR immune signal cascade reaction and inhibit the synthesis of antimicrobial peptides in vaginal mucosa. This inhibition weakens the ability of Lactobacillus to colonize, promotes the transformation of bacterial community structure toward CST IV, aggravates vaginal microecological disorder, and ultimately accelerates the process of malignant lesions of cervical epithelial cells [40].

In addition, age is a key factor closely related to vaginal microbiome composition and HPV infection. Age dynamically regulates the interaction among “virus–host–microbiota”, which not only affects the natural development of HPV infection, but also maintains homeostasis of the vaginal microecosphere. This regulatory effect is derived from three core age-related changes: hormonal fluctuations, changes in immune system maturation, and changes in personal behavior habits.

The 15–25 age group has the highest incidence of HPV infection, mainly due to the higher frequency of exposure to the virus, but this group still has a strong ability to clear the virus [41]. This phenomenon originates from a set of coordinated dual defense mechanisms: active T-cell-mediated cellular immunity can efficiently remove foreign antigens, while high estradiol levels promote glycogen accumulation in vaginal epithelium; these glycogens can support Lactobacillus to occupy the dominant position of the flora and maintain an acidic microenvironment (pH 3.8–4.5) with protective effects, so most HPV infections are only transient infections [42]. Conversely, the 35–44 age group is a key turning point. At this stage, the number of persistent HPV infections has increased significantly, and precancerous lesions have also continued to accumulate. The vaginal microecology is no longer dominated by Lactobacillus, but the flora is disordered, which is intuitively manifested by the ups and downs of Lactobacillus content and the abnormal increase in microbial diversity. These changes together weaken the ability of local innate immunity to eliminate viruses [43]. The immunosuppression caused by long-term stress and bad living habits will further hinder the body’s ability to clear the virus and make the original transient HPV infection evolve into persistent infection. Therefore, middle and late youth is a key window period for accurate early intervention [44]. After the transition from perimenopause to menopause, the level of endogenous estrogen in the human body decreases greatly, which leads to the atrophy of vaginal epithelium and the loss of glycogen. Due to the lack of glycogen as an important carbon source, the resident Lactobacillus flora finds it difficult to survive stably, the vaginal pH increases, and the complete physical and chemical barrier of the reproductive tract is also destroyed [45]. At the same time, immunosenescence appears, which is manifested by decreased virus clearance ability and impaired gene mutation surveillance function. It will not only make people more likely to be infected with HPV for the first time, but also activate latent viruses, resulting in a second peak of HPV infection rate in the elderly [46]. The key point is that the degree of susceptibility to disease caused by age is inseparable from various controllable and synergistic factors. Effects of exogenous hormones (e.g., oral contraceptives reduce Lactobacillus colonization) [47], immune tolerance during pregnancy, and unhealthy lifestyles (vaginal irrigation physically removes protective bacteria, and smoking inhibits local immunity) [48]. These factors are superimposed together to amplify age-related physiological decline and accelerate microecological imbalance. Therefore, clinical diagnosis and treatment can not only simply divide the population according to age, but also establish a multi-dimensional “age–microbiome–immunity” evaluation system. For women aged 35–44 years old, priority should be given to microecological detection, quantitative detection of Lactobacillus abundance, and flora diversity, so as to guide precise intervention methods such as probiotic planting and immune regulation. For postmenopausal women, treatment needs to be combined with local estrogen supplementation and microbiome reconstruction to block the progression of persistent infection to cancer from the root.

The estrobolome comprises gut microbial genes and their products involved in estrogen metabolism [49]. Microbial β-glucuronidases can deconjugate estrogen metabolites excreted into the intestinal lumen, allowing a proportion of the released estrogens to re-enter the circulation [50,51]. Because these enzymes occur in several gut bacterial taxa, estrobolome function cannot be inferred from the abundance of a single phylum or genus. Changes in estrogen availability may indirectly affect the vaginal microbiota. Estrogen promotes vaginal epithelial maturation and glycogen accumulation, supporting conditions associated with Lactobacillus predominance, lactic acid production, and a low vaginal pH. Altered microbial estrogen metabolism could therefore modify the vaginal environment without requiring the direct transfer of gut bacteria to the genital tract. This proposed gut–estrogen–vaginal microbiota axis remains incompletely characterized. Estrogen receptor signaling has also been implicated in HPV-associated cervical carcinogenesis. In HPV-transgenic models, estrogen and estrogen receptor-α contribute to cervical carcinogenesis in the presence of HPV oncogenes [52]. These findings provide a possible mechanistic connection, but they do not demonstrate that estrobolome-dependent changes in estrogen availability alter HPV persistence or CIN progression in humans. Direct clinical evidence for this relationship remains limited.

Human papillomavirus (HPV) has strict species specificity, and its growth and proliferation depend on epithelial cell differentiation. Its complete life cycle can only be completed in differentiated squamous epithelial cells. For a long time, this has led to a lack of natural infection models and has become a core bottleneck in mechanism exploration and drug development. In order to clarify the internal causes of HPV clearance, long-term persistent infection, and the interaction between microbiota–virus–host, and to screen vaccines and intervention targets, it is necessary to build an experimental model that can restore the real infection process, microbiota dynamic changes, immune response, and cancer progression.

So far, a variety of in vitro and in vivo infection models have been established [53,54]. These models have been widely used to study the interaction between the virus and host, viral immune escape, and the effect of the vaginal microbiome on HPV-related lesions. It is also a reliable tool for screening anti-HPV drugs, verifying the efficacy of probiotics, and optimizing vaccine design. Although the traditional model has many advantages, its shortcomings are very prominent: there is no way to completely reproduce the tissue microenvironment inside the real living body, and spontaneous genetic variation is prone to occur, which will reduce the accuracy of simulating natural HPV infection and subsequent lesion development. In addition, these models cannot construct a complete immune microenvironment, which is not conducive to the in-depth study of various immune regulation mechanisms during the development of HPV infection [55,56]. In general, all kinds of HPV infection models have their own advantages and disadvantages. In the actual scientific research work, it is necessary to use a variety of models in combination with specific research objectives, so as to more comprehensively analyze the pathogenic mechanism of HPV infection.

### 3.2. Clinical Manifestations of Vaginal Microbiome Imbalance and Their Association with HPV Susceptibility

#### 3.2.1. Clinical Correlation Between Vaginal Microbiome Dysbiosis and Persistent HR-HPV Infection or Clearance

A landmark systematic review combined with network meta-analysis [57] found that women who did not belong to the non-Lactobacillus-dominant vaginal microbiota group had an increased risk of new HPV infection, persistent HPV infection, high-grade cervical lesions, and cervical cancer. When the relative abundance of *Lactobacillus iners* is at a high level, the risk will be particularly evident. This pioneering study supports the view that the reconstruction of vaginal eubiosis is a promising method for the prevention and control of cervical cancer.

Another comprehensive and detailed systematic review and meta-analysis further explored the functional differences within the cervical vaginal microbial community. The results showed that the overall microbial diversity of the HPV-positive population was higher than that of the control group without infection. The key point is that the characteristics of the microbial community will change with the severity of the disease: in the early stage of HPV infection, the flora is dominated by *Gardnerella*; when the lesion begins to appear, the number of *Prevotella*, *Porphyromonas*, and Dialister will gradually increase. The above phenomenon shows that the structure of the flora will change according to the law of fixed time, and the development process of cervical lesions will change synchronously [58,59]. However, most of the studies included in this summary were designed in a cross-sectional study, and it was difficult to determine whether the changes in flora occurred before or after HPV infection and lesions. At the same time, the sequencing platforms used in each study are not uniform (the 16S rRNA V3–V4 region and V4–V5 region are mixed), and there are differences in bioinformatics analysis processes, which will cause batch effects and easily interfere with the conclusions of the meta-analysis. In the future, a longitudinal study using a unified sampling standard and standardized analysis system is needed to verify the dynamic changes of the flora with the course of the disease, and to build biomarkers that can judge the progression of the disease and rely on microbiome.

A systematic review and meta-analysis of observational studies further refined the association between flora and lesions: from HPV-negative people, HPV-positive people, CIN patients to cervical cancer patients, as cervical lesions continue to increase, the abundance of *Gardnerella*, *Prevotella*, *Sneathia*, and Streptococcus will continue to increase. The abundance of *Prevotella* in cervical cancer patients was significantly higher than that in HPV-negative healthy controls, while *Sneathia* was significantly increased in the CIN and cervical cancer patients [34,60]. The above results indicate that these bacteria belong to the microbial taxa potentially associated with the progression of cervical lesions, but it is not yet possible to confirm that they will be directly involved in the carcinogenesis process. Although the results of integrated statistics are highly reliable, most of these conclusions only rely on relative abundance data and lack of absolute microbial load detection as a reference, so the value of clinical application is limited. In addition, all studies included in the analysis were observational studies, and the causal relationship could not be determined. It is not clear whether the change in flora is the cause of cervical cancer or the secondary result caused by the change in local microenvironment. In the future, prospective studies are needed to combine absolute quantitative detection of microorganisms, metatranscriptomic analysis, and metabolomic analysis to clarify the actual physiological effects of these bacterial changes, as well as the sequence of bacterial changes and lesions.

Although the summarized associations are statistically reliable, the value of their application to clinical practice is not high. At present, most of the microbiome datasets only count the relative abundance of species, and there is no matching detection of absolute concentration of bacteria. Another obvious shortcoming is that the basic research is still based on the observational experimental design, which cannot determine the causal trend: whether the flora disorder is the source of cervical cancer, or the secondary change after the mucosal microenvironment is reshaped by the lesion, is still inconclusive. In order to clarify the physiological function and timing of bacterial changes, future longitudinal prospective studies need to combine the absolute quantitative methods of bacteria with multi-omics techniques including metatranscriptomics and metabolomics.

A meta-analysis of 21,446 women from 14 independent studies showed that bacterial vaginosis was significantly associated with an increased risk of HR-HPV infection. Coinfections with Ureaplasma urealyticum and Chlamydia trachomatis were also more frequent among HR-HPV-positive women [61]. The Costa Rica HPV Vaccine Trial A prospective nested cohort study was conducted to continuously track and record the dynamic changes of cervical and vaginal flora in 273 women with newly diagnosed HR-HPV infection. The results of the microbial marker analysis showed that *Lactobacillus iners* was highly correlated with virus clearance (LDA score > 4.0), and *Gardnerella* was the most associated with CIN2+ progression. Further mediation analysis showed that the high baseline *Gardnerella* abundance induced CIN2+ lesions by increasing microbial diversity, which supported a reasonable linkage mechanism at the statistical level: microecological imbalance would promote the deterioration of cervical lesions step by step [62,63].

It is worth noting that the sample size of this cohort study is small, the subjects are only from a single region, and the conclusions of the study are difficult to widely apply. Therefore, it is necessary to carry out cohort studies with larger samples and covering multiple races as well as increase the frequency of longitudinal sampling to accurately sort out the relationship between the changes in flora structure and HPV-related clinical lesions over time. Since L. iners can not only colonize in a healthy vaginal microenvironment, but also survive in a disordered microenvironment, subsequent studies cannot only stay at the level of strain analysis, but also need to carry out strain-level identification and functional gene annotation to understand its biological role in different environments.

A large retrospective cohort mechanism study of 6085 subjects, combined with experimental validation, was conducted to determine how HPV reshapes the composition of vaginal microbiota [20,64]. From the principle of action, HPV E7 oncoprotein interferes with the host NF-κB and Wnt/β-catenin signaling pathways, thereby downregulating the level of host defense peptides synthesized by epithelial cells. These endogenous peptides are the key amino acid sources to maintain the proliferation of Lactobacillus. Less supply of defensive peptides will reduce the abundance of symbiotic Lactobacillus, resulting in vaginal microbial dysbiosis. From a clinical point of view, the imbalance of flora caused by E7 will increase the risk of subsequent bacterial vaginosis to 2.35 times. The above studies have shown that there is a two-way effect between HPV and flora: after HPV changes the mucosal environment, it will in turn aggravate the flora disorder. However, most of the mechanism evidence of this study comes from in vitro experiments and K14-HPV16 transgenic mice, which cannot completely reproduce the interaction of host–microbiome–immunity in the human body. In addition, retrospective clinical analysis cannot rule out various confounding factors such as sexual behavior, contraceptive use, and antibiotic exposure. Subsequent prospective studies are needed to continuously and dynamically detect HPV infection status, defense peptide content, and microbiome composition to verify this mechanism in the human body as well as whether supplemental defense peptides can break this vicious cycle.

Regarding HPV subtype-specific interactions, a systematic review of seven studies employing metagenomic sequencing demonstrated that HPV16, the most pathogenic subtype, is associated with a distinct vaginal microbiome signature characterized by a significantly diminished relative abundance of *Lactobacillus crispatus* and an elevated colonization of *Gardnerella vaginalis*—a profile more pronounced than that observed with other high-risk types [65,66]. These findings well explain the reasons for the different carcinogenic abilities of different HPV subtypes from the microbial level. However, the number of studies specifically using shotgun metagenomic sequencing is very small, and only 7 articles qualified for this screening, which makes the statistical analysis of different HPV subtypes less convincing. At present, most of the data are from 16S rRNA gene sequencing. This technology has insufficient resolution accuracy and cannot distinguish pathogenic strains within the same strain. For example, it is impossible to distinguish between symbiotic harmless strains and pathogenic strains in G. vaginalis. Subsequent studies need to use strain-level metagenomic analysis and functional gene mapping analysis to find specific microbial genetic fragments that can cooperate with HPV16 and promote carcinogenesis.

Increasing new studies have shown that the interaction patterns between different HPV subtypes and vaginal microecology are different. A systematic review of seven metagenomic sequencing studies found that HPV16, the most carcinogenic, corresponded to unique flora characteristics. Unlike other high-risk HPV subtypes, HPV16 infection is often accompanied by a significant decrease in the abundance of *Lactobacillus crispatus* and a large proliferation of *Gardnerella vaginalis*, thus forming microbial characteristic markers that are exclusive to this subtype of lesions [65,66]. This difference in the level of flora provides a reasonable set of microbiological principles to explain the uneven malignant transformation ability of various HPV subtypes.

However, the existing research evidence still has obvious shortcomings: the number of shotgun metagenomic sequencing research samples that meet the requirements is too small, which reduces the statistical credibility of the comparison results of different subtypes of bacteria; most of the existing microbiome data are obtained by 16S rRNA sequencing, which cannot meet the resolution requirements at the strain level. The low resolution cannot distinguish pathogenic strains from symbiotic strains in the same species, such as low pathogenic *Gardnerella* and high pathogenic *Gardnerella* in the vagina. Therefore, it is urgent to carry out more accurate research. With the help of strain-level metagenomic analysis and functional gene map analysis, key microbial genetic factors can be accurately located. These factors will work together with HPV16 to promote the occurrence and development of cervical cancer.

A cross-sectional study of 624 Chinese women found that the number of *Lactobacillus iners* in HPV-infected patients increased significantly, which is a characteristic bacterial marker and also indicates that the strain is closely related to vaginal microecological disorders. Logistic regression analysis further confirmed that bacterial vaginosis and HR-HPV infection were independent risk factors for CIN lesions [67,68]. This conclusion is consistent with the conclusion of previous population studies, and also supports a view that the flora dominated by L. iners is likely to be an intermediate transition state from vaginal eubiosis to flora disorder. However, cross-sectional studies cannot determine the causal relationship. It is currently impossible to determine whether the large proliferation of L. iners makes the human body more susceptible to HPV infection, or whether HPV infection in turn promotes the proliferation of the strain. Functional genomics studies have shown that the genome structure of L. iners is streamlined and can easily change with the external environment. It can flexibly synthesize L-lactic acid and the pore-forming toxin inerolysin according to different local microenvironments. Follow-up studies need to clarify vaginal pH, nutrients, and host immune regulation, and how to regulate L. iners with both protective and pathogenic characteristics.

A meta-analysis based on public 16S rRNA gene sequencing data screened out microbial markers associated with the progression of cervical malignant lesions. The contents of Streptococcus, *Fusobacterium*, Pseudomonas and Anaerococcus were significantly increased in cervical cancer samples. On the contrary, the abundance of various Lactobacillus in the cervical tissues of healthy people was higher [69,70]. The same pattern was observed in multiple independent cohorts, indicating that this type of flora has the potential to be used as a marker based on the microbiome for cervical cancer screening. However, 16S rRNA sequencing is difficult to reflect the functional potential and metabolic activity of microorganisms. In addition, the enrichment of specific flora may only be the result of a tumor changing the local microenvironment, and not the direct induction of carcinogenesis by flora. Combined with metagenomic, metatranscriptomic, and metabolomic multi-omics analysis, it is possible to determine whether these microorganisms actively generate gene toxins, pro-inflammatory metabolites, carcinogenic signaling molecules, or simply colonize in the tumor microenvironment. A number of longitudinal studies have further confirmed that the initial vaginal microbiome features can predict the direction of subsequent HPV-related lesions. A cohort study of 141 HR-HPV-positive women showed that the long-term continuous disorder of flora structure was positively correlated with the increase in overall microbial diversity. In this cohort, *Gardnerella vaginalis* will make the disordered flora lacking dominant Lactobacillus stable for a long time, while the colonization of *Lactobacillus iners* is highly correlated with the unstable structure of Megasphaera genomosp. type 1 dominant flora [71,72]. Another prospective investigation focusing on patients with persistent HR-HPV infection further validated the potential of cervicovaginal microbial profiles to predict subsequent squamous intraepithelial lesion development, supporting microbiome-based biomarkers for cervical disease stratification after HR-HPV detection [71,73].

Although existing studies have found changes in the flora over time during the entire process of HPV infection, these studies still have obvious shortcomings. At present, most of the follow-up cycles are only 6 to 18 months, and the duration is short, which is not enough to fully cover the natural development process of HPV pathogenesis, especially the complete process of long-term evolution to high-grade cervical lesions. At the same time, the sample size of the current cohort study is generally small, and it is impossible to fully correct various confounding factors such as sexual behavior, hormone fluctuations, and combined reproductive tract infections. In the future, it is necessary to rely on large-scale, multi-center prospective cohort studies to prolong the follow-up time and increase the frequency of intensive longitudinal sampling, so as to clarify the precise timing correlation between the change of flora and the progress of the disease, and to build a clinical and reliable prediction model.

The comparison of single HPV infection and multiple HPV infection provides a new idea for the study of changes in flora function. A cross-sectional study of 508 Indian women showed that patients with cervicitis were significantly more likely to develop BV infection, epithelial cell abnormalities, and HPV infection than healthy controls without any symptoms [74,75].

With the help of an optimized bioinformatics analysis process, the high-precision detection of cervical and vaginal microbiome strains can now systematically evaluate the relationship between flora composition, HPV infection status, and CIN progression. This analysis method improved the species resolution accuracy to the genus level, and found a rule that could not be found by previous low-resolution 16S rRNA sequencing: different strains have different associations with HPV infection and cervical lesion severity [76,77]. The strain-level resolution achieved in this study is an important methodological advance, because only the genus level is divided, which often masks the distinct roles of closely related strains and strains. However, a biological information analysis process that relies on short-reading long-sequencing data to achieve strain resolution is highly dependent on the reference database. The reference database for small populations and remote geographical populations is not perfect, which will cause analysis bias. The vaginal microbiomes of women in low- and middle-income countries, where the cervical cancer burden is highest, remain significantly underrepresented in existing reference databases. Investments in population-specific microbial genome sequencing and culturomics efforts are needed to ensure equitable applicability of species-level microbiome diagnostics.

Emerging in vitro studies have explored the direct regulatory effects of specific *Vaginal bacterial* species on the expression of HPV oncogenes. A key mechanism study [78] confirmed that *Lactobacillus crispatus* and *Lactobacillus gasseri* can significantly inhibit the expression of E6 and E7 genes in HPV16-infected cells and reduce the production of oncoprotein. On the contrary, *Gardnerella vaginalis* and Megasphaera micronuciformis significantly increased the expression levels of E6 and E7 genes, while the contents of p53 and pRb in cells decreased, and the proportion of cells entering the S-phase increased. A review of the literature [79] further sorted out the four action paths of cervical vaginal flora to promote HPV-related cervical cancer: (i) modulation of genital inflammation and immune responses, (ii) alteration of HR-HPV oncogene expression and oncoprotein production, (iii) regulation of oxidative stress and DNA damage, and (iv) induction of metabolic rewiring. These in vitro experimental results confirm that there is a direct correlation between specific strains and HPV oncogene activity, and also reasonably explain the correlation observed in various clinical epidemiological studies from a biological perspective. However, a single strain culture experiment cannot restore the complex ecological interactions under the coexistence of multiple microorganisms. The metabolic cross-supply, quorum sensing, and competitive exclusion between bacteria will change the characteristics of bacterial activity. In addition, the concentration of bacterial metabolites and the multiplicity of infection used in in vitro experiments are not consistent with the real physiological environment of the cervix and vagina. If a three-dimensional organoid model that integrates a variety of bacteria, host epithelial cells, and immune cells and matches the physiological oxygen concentration gradient is built, an experimental platform that is more suitable for clinical transformation can be created to facilitate in-depth disassembly of the host–microbiome–virus interaction mechanism.

#### 3.2.2. From Observation to Intervention: Modulating the Vaginal Microbiome to Eliminate HPV

The core goal of clinical transformation is to develop effective therapeutic interventions. At present, the intervention programs targeting the microbiome are mainly divided into several categories: probiotics, prebiotics, vaccines prepared using engineered probiotics as carriers, and traditional Chinese medicine therapies that rely on mature Chinese medicine theory and continue to have new pharmacological research evidence [80] (see Figure 2).

The upper panel contrasts a Lactobacillus-dominant vaginal microbiota, typically associated with a low vaginal pH and an intact mucosal barrier, with dysbiosis characterized by Lactobacillus depletion, anaerobic bacterial enrichment, and impaired barrier function. These microbial states have been associated with HR-HPV persistence and clearance, but the direction of the relationship remains unresolved. Microbiome studies commonly consider microbial composition alongside participant characteristics, HPV genotype and infection status, vaccination history, and longitudinal outcomes. The lower panel summarizes three investigational approaches: probiotic or prebiotic supplementation to restore Lactobacillus predominance; engineered probiotic vectors for the mucosal delivery of HPV antigens; and Jiawei Ermiao Granules for the modulation of vaginal microecological parameters. Engineered probiotic vaccines remain at the preclinical stage. Evidence for probiotics, prebiotics, and Jiawei Ermiao Granules is based on limited clinical studies and is not sufficient to support routine use. Green and red shading denote healthy and dysbiotic vaginal microecology, respectively; arrows indicate the direction of effect or risk change.

**(1)**  **Probiotic and Prebiotic Interventions**

Probiotic interventions aimed at the reconstruction of Lactobacillus-dominated vaginal microecology have become a promising adjuvant therapy in the treatment of HPV infection [81]. A meta-analysis confirmed that probiotic adjuvant therapy can effectively improve the HPV-negative conversion rate in patients with cervical intraepithelial neoplasia [82]. A pilot randomized trial selected 117 women with both bacterial vaginosis and HPV infection. The study found that the use of Lactobacillus rhamnosus BMX 54 for a 6-month vaginal administration intervention was better than a 3-month short course of treatment. HPV-related cytological lesions are more likely to improve, and the virus clearance efficiency is also higher [83]. Another multi-center randomized placebo-controlled trial included 62 subjects with newly diagnosed HPV infection. Oral supplementation of *Lactobacillus crispatus* M247 could significantly improve the therapeutic effect: the HPV clearance rate increased from 31.8% to 60.0%, and the proportion of people with normal Pap smear increased from 71.4% to 83.3%. The probiotic intervention can also promote the transformation of the flora into CST I, that is, the vaginal typing with L. crispatus as the dominant bacteria [84,85]. In a 12-week randomized placebo-controlled trial, Lactiplantibacillus plantarum Probio87 reduced the relative abundance of *Gardnerella vaginalis*, *Fannyhessea* vaginae, and Bilophila in the intestinal flora of HPV-positive women, while increasing the abundance of Anaerotignum lactatifermentans and Blautia faecis. The results preliminarily proved that the gut–vaginal axis can be used for HPV intervention conditioning [86,87]. In addition, a prospective controlled pilot study of 100 women with HR-HPV infection showed that intravaginal transplantation of *L. crispatus* chen-01 reduced HPV viral load, increased viral clearance, and alleviated vaginal inflammation without evident adverse reactions [88,89]. Collectively, these studies suggest that probiotic interventions may facilitate HPV clearance and restore vaginal microbial homeostasis. However, substantial heterogeneity in probiotic strains, doses, administration routes, and treatment durations prevents the identification of an optimal regimen. The number of subjects in many related clinical trials is less than 100, and the follow-up period is relatively short. It is difficult to judge whether the probiotics can stably remove the virus for a long time and the long-term effect on the development of CIN lesions. In addition, most studies only observed HPV-negative conversion, and did not use the regression of lesions confirmed by pathological biopsy as a criterion. Therefore, in order to list probiotics as a routine adjuvant therapy for HPV-related cervical diseases, it is also necessary to carry out large-scale, multi-center, randomized placebo-controlled trials, unify the specifications of probiotics, prolong the follow-up time, and adopt clinical efficacy evaluation indicators.

**(2)**  **Engineered Probiotic Vector Vaccines**

At present, the new targeted therapy program focuses on the delivery of the HPV vaccine using engineered probiotic carriers. Oral candidate vaccine BLS-M07, recombinant Lactobacillus casei was used to present the HPV16 E7 antigen. In a phase I/IIa dose escalation clinical trial involving CIN3 patients, 6 out of the 8 subjects were cured after inoculation, with an effective rate of 75%. The vaccine can induce strong systemic immunity in the body, which is manifested by a significant increase in the content of HPV16 E7-specific antibodies in serum, and a significant increase in the Th1 cell immune response targeting E7 (*p* < 0.05) [90].

GLBL101c represents another *L. casei*-based oral vaccine designed to express the HPV16 E7 protein. Its therapeutic efficacy was validated in a randomized, double-blind, placebo-controlled phase IIb trial comprising 40 patients with HPV16-positive CIN2 lesions. Within 16 weeks of intervention in the vaccine group, the lesions of two subjects completely subsided, and the body produced an antigen-specific Th1 immune response [91,92]. A systematic review of related research in the past 20 years was summarized, and the progress of a HPV vaccine based on lactic acid bacteria in preclinical research and clinical trials was reviewed. It was pointed out that this kind of vaccine can play an immune role and therapeutic effect based on the gut–vaginal axis, and has great potential [93].

Engineered probiotic carrier vaccines, which combine microbiome research and immunotherapy technology, are an emerging treatment method. Such vaccines have practical advantages such as convenient oral administration, affordable price, and stable safety. However, the phase IIb clinical trial of GLBL101 c was not effective, exposing the current shortcomings of the vaccine: only 2 out of the 20 subjects achieved complete regression of the lesion, the cure rate was only 10%, and the clinical efficacy was not ideal.

Insufficient immunogenicity is the primary problem hindering the popularization of such vaccines. It is difficult for an oral probiotic vaccine to stably induce a strong, long-term and mucosal T cell immune response in the local microenvironment of the cervix. Therefore, it is necessary to optimize a number of core conditions, including antigen expression intensity, adjuvant matching, and delivery carrier system. In addition, the engineered probiotics introduced exogenously will have ecological competition with the vaginal native flora, it is difficult to stabilize colonization, and it will not be able to continuously transport antigens in the lesion. Follow-up studies can improve the above problems to improve the therapeutic effect: synchronous co-expression of immune stimulating molecules can enhance the immunogenicity of vaccines, and optimizing the dosage regimen can standardize the treatment process; at the same time, the combination of a probiotic vaccine, immune checkpoint inhibitors, and conventional clinical therapy is expected to produce a synergistic anti-tumor effect, and also provides a feasible idea for the optimization of a comprehensive treatment plan.

**(3)**  **Vaginal Microbiota Transplantation (VMT)**

In addition to probiotic intervention and vaccine therapy, vaginal microbiota transplantation (VMT) has arisen as a novel therapeutic option for vaginal microecological disorders. Based on the experience of mature fecal microbial transplantation technology, a systematic review comprehensively summarized the latest research progress and clinical value of VMT in the repair of vaginal dysbiosis [94]. A large number of preclinical animal experiments and human clinical studies have verified that VMT is a safe and effective approach to reconstruct the vaginal flora with Lactobacillus as the dominant bacteria and alleviate various clinical symptoms caused by recurrent bacterial vaginosis. More and more research data also show that VMT may be able to reduce the body’s susceptibility to sexually transmitted diseases, further broadening its future clinical application.

Different from the supplementation of a single strain, VMT directly transplants a complete set of microbial communities to fundamentally reshape the micro-ecological environment. In theory, transplanting a complete set of flora combinations with normal physiological functions can achieve a more stable and longer colonization in the vagina than placing a single probiotic strain alone. However, the evidence supporting the clinical use of VMT is still relatively shallow, and most of the related studies are only small-scale case observations and pre-experiments. At present, there are several major problems hindering the standardized clinical implementation of VMT. First, a strict donor screening system must be established to prevent the spread of pathogens, and it is also necessary to investigate hidden pathogens that cannot be detected by conventional detection methods. Second, sample processing, cryopreservation, and clinical transplantation operations need to develop a unified standard process. Third, it is necessary to improve the unified donor screening criteria. Screening should not only simply screen out pathogenic bacteria, but also accurately screen out “high-quality donors” with excellent flora quality. Fourth, long-term safety follow-up monitoring must be carried out to prevent the horizontal transmission of drug resistance genes and virulence factors among the flora. It is worth mentioning that there is no clinical evidence to prove that VMT is effective for HPV-related cervical lesions, and there is no clinical trial to explore whether VMT can accelerate HPV-negative conversion and curb the continuous progress of CIN, which is also an urgent research gap in this field [95].

#### 3.2.3. Clinical Evidence of Traditional Chinese Medicine in Regulating the Vaginal Microbiome

Traditional Chinese medicine (TCM) has gradually become a potential intervention to regulate vaginal microecology and accelerate the negative conversion of high-risk HPV. More and more clinical studies have confirmed its therapeutic value. Exogenous supplementation of probiotics is only to introduce one or more beneficial strains into the body, but traditional Chinese medicine therapy is different. It takes “strengthening the body resistance and eliminating pathogenic factors” as the core idea, focusing on restoring the homeostasis of the human body’s own internal environment, relying on internal regulation to improve the local vaginal microenvironment, and reconstructing a balanced and stable flora structure. A prospective clinical trial explored the therapeutic effect of Jiawei Ermiao Granules (JWEMGs) in patients with persistent HR-HPV infection [96]. In this study, 65 patients with syndrome of downward flow of damp-heat were included, and two courses of oral administration were continuously carried out for 3 months. The final statistics showed that the overall negative conversion rate of HR-HPV was 73.85%, of which 55.38% were completely cleared and 18.46% were partially cleared. At the same time, the drug can significantly improve vaginal cleanliness, pH value, Nugent score, Donders score, and other vaginal microecological indicators. The researchers carried out metagenomic sequencing on the paired vaginal lavage fluid samples of 33 patients before and after treatment. The results showed that the vaginal microbiome flora structure of the subjects was significantly improved after the intervention of Jiawei Ermiao Granules. LEfSe analysis verified *Lactobacillus crispatus* as the dominant microbial biomarker post-treatment, with an LDA score exceeding 4.0. Microbial community typing further confirmed a clear transition from CST III/IV microecological patterns to *L. crispatus*-predominant CST I, a shift closely correlated with viral clearance and improved vaginal microecological status [96]. A supporting metabolomic study further uncovered the metabolic regulatory mechanisms responsible for JWEMGs’ therapeutic efficacy against persistent HR-HPV infection.

Integrated targeted and untargeted metabolomic analyses have uncovered widespread metabolic reprogramming triggered by JWEMG intervention in patients with persistent HPV infection. This research screened core metabolic biomarkers closely linked to treatment responsiveness, providing solid mechanistic evidence to explain how TCM regimens exert therapeutic effects on HPV-associated cervical disorders [97,98]. Collectively, current clinical and mechanistic studies on TCM treatment carry important research significance. Beyond validating the clinical capacity of TCM to eliminate persistent HR-HPV infection, existing research also systematically interprets the modern biological connotation of the TCM principle of reinforcing healthy qi and eliminating pathogens. Such interpretation is realized through two corroborating mechanisms: remodeling the disordered vaginal microbial community and enhancing host immune function [27].

However, several limitations inherent to this study should be addressed to guide future research [99]. First, the study was a single-center, single-arm trial without a control group. Although it is suitable for preliminary exploratory research, it is impossible to rule out the placebo effect and statistical regression bias. To confirm the true therapeutic effect of JWEMG, rigorous randomized, double-blind, placebo-controlled trials are needed. Second, this experiment only included 65 subjects, and the subjects were limited to patients with damp-heat damp-heat pouring downward syndrome. The research conclusions were not representative enough. The current research results cannot be applied to other TCM syndromes or a wider range of persistent HR-HPV infection. Third, at present, the academic community believes that the ALPK1/NF-κB pathway is the key target for JWEMG to exert its efficacy, but the mechanism conclusion only relies on in vitro experiments, and also needs to be further verified by the in vivo HPV infection model to consolidate the mechanism-related conclusions. Fourth, JWEMG is a compound Chinese medicine preparation, containing a large number of active substances. It is not clear which active ingredients dominate the intestinal and cervical microecological remodeling and immune regulation. In the future, it is necessary to carry out biological activity-oriented component separation and identification research to determine the effective substances that play a central role in this prescription. Fifth, there is a lack of head-to-head comparative studies against conventional first-line treatments, such as imiquimod topical therapy and surgical resection. Moreover, the synergistic effects of combined JWEMG and probiotic or routine clinical interventions remain unexplored. Relevant comparative and combination trials are needed to establish the optimal clinical application paradigm for JWEMG in treating persistent HPV infection. Finally, existing studies only adopted a 6-month follow-up period, which is insufficient to evaluate the long-term durability of viral clearance and microecological recovery. Extended observational studies are required to assess sustained therapeutic outcomes and monitor the long-term risk of cervical lesion recurrence. Collectively, resolving the above research gaps through large-sample, multicenter, and well-controlled clinical trials will help solidify the clinical evidence of JWEMG for HPV-related cervical disorders. Such efforts will also promote the standardized clinical integration of holistic TCM therapeutic strategies into conventional cervical disease management systems (see Table 1).

Evidence status was graded by study design using the Oxford Center for Evidence-Based Medicine (CEBM) levels; journal impact factor and quartile were not considered when rating evidence quality. No listed intervention has yet been validated as standard treatment for HR-HPV infection or cervical disease.

## 4. Vaginal Microbiome and Cervical Cancer: From Bench to Bedside

### 4.1. Microbial Dysbiosis and Persistent HPV Infection with Precancerous Lesions

Cervical HPV infection, sustained viral persistence, and precancerous lesions are closely correlated with non-Lactobacillus-predominant vaginal dysbiosis, which differs substantially from healthy vaginal microecology. This dysbiotic profile is typified by a dominant CST IV microbial pattern, elevated microbial α-diversity, and the overgrowth of multiple anaerobic bacterial genera. Representative dysbiosis-associated microbes include *Gardnerella vaginalis*, *Prevotella*, *Fannyhessea vaginae* (previously named *Atopobium vaginae*), *Sneathia*, Megasphaera, Peptostreptococcus, and Anaerococcus [26,99]. As cervical disease progresses from normal cervical epithelium to LSIL, HSIL, and cervical cancer, Lactobacillus dominance appears to decline, whereas anaerobe-dominated communities become increasingly prevalent [80]. A meta-analysis of 17 studies found that *Sneathia* abundance was significantly higher in patients with CIN or cervical cancer than in HPV-negative individuals, while *Prevotella* was enriched in cervical cancer [60]. Consistent with these findings, a retrospective investigation also observed increased vaginal microbial diversity in patients with HPV positivity, CIN, and cervical cancer relative to individuals with normal cervical cytology classified as no intraepithelial lesion or malignancy (NILM). Additionally, *Gardnerella* and its affiliated microbial taxa were identified as key microbes closely linked to the progression of cervical lesions [100]. A standardized meta-analysis further confirmed a consistent transition from Lactobacillus-dominated to anaerobe-dominated communities in cervical cancer (see Figure 3) [101].

The figure compares microbial community structure, vaginal ecological features, and clinical context across HPV-negative controls, HR-HPV infection, persistent HR-HPV infection and/or LSIL, HSIL (CIN2–3), and invasive cervical cancer. HPV-negative controls often show a low-diversity, Lactobacillus-dominant community and a low vaginal pH. Persistent HR-HPV infection, HSIL, and cervical cancer have been associated with lower Lactobacillus abundance, a greater frequency of CST IV or other non-Lactobacillus-dominant communities, higher vaginal pH, increased microbial diversity, and the enrichment of anaerobic taxa. These features vary across populations, sampling sites, and analytical methods. The categories shown are not a fixed temporal sequence: HR-HPV may clear, and precancerous lesions may persist, regress, or progress. The figure summarizes group-level associations and does not establish that dysbiosis causes HPV persistence or cervical disease progression. The color gradient from green to red reflects progression from a healthy, Lactobacillus-dominant state to invasive cervical cancer.

#### 4.1.1. Transition at the Cin1 Stage

HR-HPV infection and CIN1 are associated with early alterations in the vaginal microbiota. The overall abundance of beneficial Lactobacillus species, including *L. crispatus*, *L. gasseri*, and *L. jensenii*, undergoes a noticeable reduction [102,103]. While the relative abundance of Lactobacillus species decreases, the vaginal microbial diversity increases, as does the proliferation of conditional pathogenic bacteria and anaerobic bacteria such as *Gardnerella* spp. and *Prevotella* spp. [104,105]. Such changes in the structure of the flora indicate that CIN1 is an early transitional stage in the process of cervical lesions. At this stage, the vaginal microenvironment, which was originally dominated by Lactobacillus and had a protective effect, will gradually transform into a microenvironment with a high diversity of flora and obvious pro-inflammatory characteristics. It is generally believed that this kind of microecological changes will form a suitable environment in the cervix, which will contribute to the persistent infection of HPV and promote the deterioration of subsequent lesions.

#### 4.1.2. Further Progression at Cin2 and Cin3 Stages

When low-grade cervical lesions gradually develop to CIN2 and CIN3, the degree of vaginal microecological disorders will be significantly aggravated. HSIL/CIN2-3 frequently shows further depletion of Lactobacillus, particularly *L. crispatus*, and the enrichment of diverse anaerobic or opportunistic taxa, including *Gardnerella*, *Prevotella*, *Sneathia*, *Atopobium*/*Fannyhessea*, and *Ureaplasma* spp. [102,103,105]. Although the overall abundance of Lactobacillus continues to decline as the disease progresses, L. iners-dominant vaginal communities have been associated with microbial instability and persistent HPV infection. Separately, tumor-resident *L. iners* has been linked to chemoradiation resistance in cervical cancer, although these findings should not be directly extrapolated to vaginal community composition [66]. The harmful anaerobic bacteria in high-grade CIN lesions will be further amplified, and the number of *Gardnerella vaginalis*, *Prevotella*, and Microbacterium will increase significantly. Many kinds of anaerobic opportunistic pathogens, such as *Atopobium vaginae*, *Ciliated bacteria*, *Ureaplasma urealyticum* and so on, proliferate in patients with advanced CIN and cervical cancer. Higher-grade lesions are generally associated with greater microbial diversity, although the magnitude and stage-specific pattern vary among cohorts [102,105]. A cross-sectional study on the changes of flora in different CIN grades confirmed that the disorder of flora was stepwise aggravated, and that the structure of vaginal flora would be dynamically reshaped with the severity of the lesion [106]. In general, these findings indicate progressive ecological disruption across advanced cervical disease states, but longitudinal studies are required to determine whether dysbiosis drives lesion progression or develops as a consequence of the altered cervical microenvironment (Figure 4).

The schematic compares HPV-negative healthy individuals with those who have HR-HPV infection, persistent HR-HPV infection and/or CIN1, CIN2–3, or cervical cancer. Studies of CIN2–3 and cervical cancer commonly report reduced Lactobacillus abundance, greater microbial α-diversity, elevated vaginal pH, and enrichment of anaerobic taxa, including *Gardnerella*, *Sneathia*, and *Prevotella*. Greater genital tract inflammatory activity has also been reported in high-grade lesions and cervical cancer. These findings vary across cohorts and analytical methods. The arrangement illustrates disease-associated patterns rather than an inevitable sequence of progression or a causal effect of dysbiosis. Open circles denote α-diversity, green rods denote Lactobacillus, red/purple rods denote anaerobic taxa (*Gardnerella*, *Sneathia*, *Prevotella*), triangles denote vaginal pH, and arrows indicate an increase or decrease; the shading gradient from light to dark reflects progression from healthy mucosa to cervical cancer.

### 4.2. Mechanisms by Which Dysbiosis Promotes the Occurrence and Progression of Cervical Cancer

Vaginal microbial dysbiosis is not just an incidental change that occurs passively during the development of cervical cancer. More and more clinical and mechanistic studies have shown that unbalanced vaginal flora actively promotes cervical tumorigenesis, relying on three interrelated regulatory pathways: local microenvironmental remodeling, host metabolic reprogramming, and epigenetic modification of virus and host gene expression reprogramming, and epigenetic modification of both viral and host gene expression [107]. It is important to note that vaginal dysbiosis and HPV infection are two-way relationships. The disordered micro-ecological environment will create a suitable living environment and promote sustained HPV persistence; in turn, HPV infection can also change the structure of vaginal flora. In detail, viral oncoproteins disrupt cervical epithelial integrity, indirectly causing a large number of pathogens to multiply, making microecological imbalance more serious (see Figure 5) [27,108,109,110].

In a Lactobacillus-dominant environment, lactate production helps maintain a low vaginal pH. Bacteriocins, nutrient competition, and competition for epithelial adhesion sites can restrict pathogen colonization. Hydrogen peroxide production may provide additional antimicrobial activity under specific strain and environmental conditions. Lactobacilli are also associated with epithelial barrier integrity and the regulation of local inflammatory responses, both of which are relevant to mucosal antiviral defense.

A reduction in Lactobacillus abundance may coincide with lower lactate concentrations, higher vaginal pH, and anaerobic bacterial enrichment. Microbial metabolites and epithelial barrier impairment in this setting have been linked to altered host metabolism and immune regulation. Reported mechanisms include reduced CXCL14 expression, impaired T-cell responses, and persistent Toll-like receptor signaling. HPV E6 and E7 disrupt cell-cycle control by targeting p53 and pRb, respectively. Persistent infection is also accompanied by changes in viral and host epigenetic regulation. Chronic inflammatory signaling and reactive oxygen species can increase DNA damage and genomic instability, providing conditions that may support malignant transformation. The figure combines mechanisms supported by different types of evidence and does not imply that each pathway occurs sequentially or has been established in patients.

#### 4.2.1. Local Microenvironmental Changes

The decrease in the number of beneficial Lactobacillus colonization will reduce the secretion of vaginal lactic acid, resulting in an increase in local pH. Such microenvironment changes will create suitable growth conditions for the proliferation of anaerobic pathogens [111,112]. The characteristic flora of CST IV can secrete enzymes that can degrade mucins such as sialic acid enzymes, which will destroy the tight junction structure of the epithelium and weaken the protective ability of the cervical mucosal barrier. When this tissue structure is damaged, HPV can more easily invade the basal layer of the epithelium, eventually causing persistent viral infection [113]. In addition, anaerobic bacteria and the bioactive substances produced by them can activate the body’s pattern recognition receptors, especially Toll-like receptors. After the immunity is activated, pro-inflammatory cytokines such as IL-1β, IL-6, IL-8, and TNF-α will continue to be released, leaving the cervix in a low-grade chronic inflammatory state for a long time [108]. Long-term inflammation can cause reactive oxygen species accumulation, DNA damage, genomic instability, and DNA repair mechanism disorder. At the same time, it also inhibits chemokines such as CXCL14, weakens the function of antigen-presenting cells, hinders the recruitment and activation of T cells, helps HPV to achieve immune escape, promotes the continuous development of cervical lesions, and eventually canceration [27,114]. Various experimental results also show that protective lactobacilli can enhance the body’s antiviral ability, and pathogenic bacteria associated with flora disorders can induce a strong pro-inflammatory response and destroy cervical epithelial homeostasis and immune homeostasis [115].

#### 4.2.2. Metabolite Alterations

The abnormal metabolism of vaginal microenvironment caused by flora disorder is closely related to the continuous infection of HPV and the continuous development of cervical cancer. The dominant beneficial bacteria *Lactobacillus crispatus* and *Lactobacillus jensenii* secrete D-lactic acid to maintain vaginal metabolism stability and build up immune protection. In contrast, *Lactobacillus iners* mainly synthesizes L-lactic acid, which has a weak protective effect against bacterial flora disorders and virus invasion [116]. Studies have confirmed that D-lactic acid has anti-tumor effects, which can regulate YAP1 signaling activity, regulate the renewal cycle of cervical stem cells, and curb the development of cancer [117]. In addition, erucic acid secreted by L. crispatus can activate the PPAR-δ pathway and induce ferroptosis in cervical cancer organoids, and found a new anti-cervical cancer mechanism of probiotics [118].

Under the disordered state of Lactobacillus-deficient flora, the content of vaginal biogenic amines increased significantly, and the pH value of the vagina increased, which not only facilitated the proliferation of BV-related bacteria, but also increased the virulence of pathogenic bacteria and helped pathogenic bacteria avoid the clearance of innate immunity [66]. Excessive accumulation of biogenic amines in the vaginal microenvironment can induce long-term chronic inflammation. This persistent inflammation is a key pathogenic factor that will keep HPV in the body for a long time and accelerate the malignant development of cervical lesions [66]. When cervical intraepithelial neoplasia (CIN) develops to CIN2 and CIN3, the metabolic pathways of the flora will change significantly: the healthy flora is mainly enriched in amino acid and carbohydrate metabolism, and after the lesion, it will turn to nucleotide metabolism and peptidoglycan synthesis pathway enrichment. Anaerobic bacteria such as *Gardnerella vaginalis*, *Bifidobacterium longum*, *Vaginal bacteria*, *Prevotella saccharolytica*, *Prevotella timone*, *Streptococcus lactis*, and *Streptococcus anginosus* are the main flora that promote this metabolic transformation. The metabolic remodeling caused by the disorder of vaginal flora has a dual pathogenic effect, which not only promotes the persistent infection of HPV, but also promotes the development of malignant cervical lesions. Elevated levels of nucleotides and peptidoglycan synthesis represent the rapid proliferation of disordered pathogenic bacteria. They are the core components of microbial nucleic acid and the cell wall. This type of metabolic hyperfunction provides the necessary material basis for long-term HPV colonization and the deterioration of lesions. At the same time, a large amount of accumulated bacterial peptidoglycan can be used as a ligand for pattern recognition receptors such as TLR2, which continuously activates the inflammatory response, creates a long-term tumor microenvironment, and accelerates the progression of cervical cancer [107]. HPV-encoded E6 and E7 oncoproteins are potent inducers of host metabolic reprogramming. They enhance the intensity of glycolysis, promote the Warburg effect, and inhibit mitochondrial oxidative phosphorylation, making host cells highly dependent on glucose metabolism to generate lactic acid. The imbalance of vaginal microecology will further aggravate this metabolic disorder through a variety of synergistic ways: pathogenic anaerobic bacteria will compete with cervical cells for limited glucose, release metabolic by-products to interfere with the normal use of glucose by cells; the continuous reduction in beneficial Lactobacillus and the proliferation of anaerobic bacteria will also change the type and concentration of lactic acid in the cervical microenvironment. Lactic acid can be efficiently metabolized by tumor cells with the help of MCT-1 and MCT-4 transporters. It is an important energy substrate for tumor cells. This process maintains an abnormal Warburg phenotype and is closely related to the poor prognosis of cervical diseases [119]. The upregulated glycolytic metabolism can provide sufficient energy and biosynthetic raw materials to support the unlimited proliferation of tumor cells. A large amount of lactic acid secretion will aggravate the acidification of the local cervical microenvironment. The acidic microenvironment can further stabilize HPV persistent infection, promote tumor growth, invasion and metastasis, and help cancer cells evade immune surveillance [120].

In sharp contrast, *Lactobacillus iners* has always been a controversial strain in vaginal microecology-related research. Different from recognized protective strains such as L. crispatus, L. iners mainly synthesizes L-lactic acid, and its metabolic stability and mucosal protective effect are worse [121,122]. It produces inerolysin, a cytolytic toxin that is structurally and functionally similar to the vaginal lysozyme produced by *Gardnerella vaginalis*. The expression of inerolysin may impair cervical epithelial integrity, thereby enhancing bacterial adhesion and the invasive capacity of opportunistic pathogens within the vaginal microenvironment. However, its functional effects appear to depend on the surrounding microbial community and host immune status. Accordingly, L. iners-dominated communities are often considered an unstable intermediate state between Lactobacillus-dominated eubiosis and CST IV dysbiosis and have been associated with persistent HPV infection, bacterial vaginosis-like dysbiosis, precancerous lesion progression, and an increased risk of certain treatment-related adverse effects [120,123].

#### 4.2.3. Effects on HPV Oncogene Expression and Host Epigenetics

Persistent HPV infection is a fundamental condition enabling the continuous expression of viral E6 and E7 oncoproteins. Specific Lactobacillus strains are able to downregulate E6/E7 expression, curtail oncoprotein accumulation, and restrain the proliferation of cervical tumor cells. On the contrary, microbial taxa correlated with bacterial vaginosis, such as *Gardnerella vaginalis* and Megasphaera, exert the opposite pro-oncogenic effects [124,125]. In vitro experiments showed that Lactobacillus species and dysbiosis-associated bacteria differentially regulated E6 and E7 oncoproteins and the corresponding tumor suppressors p53 and pRb in HPV16-infected cells [126]. Specifically, *Lactobacillus crispatus* and *Lactobacillus gasseri* markedly suppress the transcription and protein synthesis of E6 and E7 oncogenes. In contrast, *Gardnerella vaginalis* and *Micromegasphaera micronuciformis* have carcinogenic effects, which can significantly upregulate the transcription level of E6/E7 and increase the content of E7 oncoprotein, thereby enhancing the carcinogenic effect of the virus. *Lactobacillus crispatus* and *Lactobacillus gasseri* can strongly inhibit the transcription process of E6 and E7 and reduce the content of corresponding oncoproteins. *Gardnerella vaginalis* and *Micromegasphaera micronuciformis* play a role in promoting cancer: the two pathogens activate E6/E7 transcription and upregulate expression of the E7 oncogene. At the level of host tumor suppressor genes, p53 and pRb were significantly downregulated in cells co-cultured with *Micromegasphaera micronuciformis*, and the proportion of cells entering the S-phase was significantly increased. The above phenomena directly indicate that different strains can disrupt the normal cell cycle regulation mechanism of host cells by regulating the expression of HPV oncogenes.

Additionally, dysbiosis can rely on epigenetic alterations to affect host tumor suppressor genes, inflammation-related pathways, and viral integration processes. In addition to disrupting vaginal microecology and remodeling cell metabolism, vaginal microbial dysbiosis also carries out the epigenetic modification of HPV and the host transcriptome to promote cervical malignant transformation. Metabolites secreted by symbiotic microorganisms, especially short-chain fatty acids such as butyrate, can inhibit histone deacetylases; this process will further change the degree of chromatin opening and regulate the transcriptional activity inside the cervical epithelial cells. At the same time, dysbiosis disrupts one-carbon metabolic flux, reduces the supply of methyl donors, and ultimately alters genome-wide DNA methylation landscapes. Collectively, such epigenetic rewiring sustains persistent HPV colonization: it blunts endogenous antiviral defenses, compromises the physical integrity of cervical epithelial barriers, and fosters an inflamed, immune-suppressive mucosal niche. To date, direct empirical data that definitively tie microbe-triggered epigenetic remodeling to the advancement of cervical neoplasia are still scarce. Even so, existing published work constructs a biologically reasonable mechanistic cascade underlying this association.

Therefore, epigenetic reprogramming is a promising pathway connecting vaginal dysbiosis and cervical cancer, but the current mechanism of this pathway has not been thoroughly studied. At present, it is urgent to carry out prospective cohort studies, combined with in vitro and in vivo functional experiments, to verify the causal relationship between epigenetic alterations caused by flora and the progression of lesions, and to accurately locate the core microbial metabolites and host molecular targets that promote this series of signal transduction.

More and more studies have shown that cervical vaginal flora plays a key role in the process of cervical cancer. Although vaginal dysbiosis is associated with various pathological changes, the molecular mechanism of how the disordered flora acts on the apparent modification of HPV is still unclear. The decrease in Lactobacillus and the excessive proliferation of anaerobic bacteria will maintain the mucosal inflammation for a long time, upregulate the expression of DNA methyltransferase, change the composition of local metabolites, and then create an environment suitable for the occurrence of HPV DNA methylation abnormalities. Dysbiosis-associated immune suppression may also facilitate HPV integration, which is accompanied by extensive epigenetic reprogramming of the viral genome. Future studies combining vaginal microbiome profiling with HPV-specific methylation sequencing in CIN2 cohorts are needed to determine whether particular microorganisms or their metabolites regulate HPV methylation through host epigenetic enzymes. Integrating microbial signatures with HPV methylation profiles may also facilitate non-invasive risk stratification and help predict CIN2 regression or progression.

### 4.3. Clinical Translation

#### 4.3.1. Diagnosis and Prognosis Assessment

(1) Early diagnosis and screening:
  ① SNV (Single Nucleotide Variant) biomarker

Driven by ongoing advances in microbiome research, analytical techniques have evolved progressively from traditional 16S rRNA gene sequencing to high-resolution metagenomic sequencing and strain-level microbial profiling. In this context, microbial single-nucleotide variants (SNVs) have been recognized as promising biomarkers for cervical disease screening, as they can precisely reflect strain-specific microbial alterations during disease progression. A recent metagenomic investigation enrolled a total of 151 clinical samples, covering healthy individuals and patients diagnosed with HPV infection, cervical intraepithelial neoplasia (CIN), or cervical cancer. Hu et al. showed that microbial SNV profiles reflected both community composition and disease-associated differences in microbial functional capacity and strain adaptation. Quantitative comparison revealed markedly higher microbial SNV counts in the cervical cancer group than in the HPV-infected group (14,892 vs. 9847), with significant disparities in both SNV quantity and mutation composition across groups. These differences at the genomic level indicate that during the whole process of the continuous development of cervical lesions, the vaginal microbial genome will undergo dynamic changes that match the lesion stage. This study also screened out genes with obvious SNV mutations and key functions in the samples. The genetic variation of *Gardnerella vaginalis* was significantly enriched in CIN and cervical cancer patients. Among them, the CYJ64_RS02490 gene has a higher incidence of nonsense mutation in cervical cancer tissues, which is likely to destroy the expression and physiological function of CadD family cadmium resistance transporters. The HMPREF9216_RS00340 gene is responsible for encoding NUDIX hydrolase involved in folic acid metabolism. The genetic variation of this locus is likely to reshape the metabolic characteristics of the flora and then affect the cervical microenvironment. In Streptococcus agalactiae, the high-frequency mutation of AMM49_RS05120 gene encoding tetracycline-resistant ribosomal protection protein Tet (M) is associated with the prevalence of cervical cancer, but the exact pathogenesis of this mutation is still unclear. Functional enrichment analysis further confirmed that different stages of lesions corresponded to different changes in the flora pathways: the flora of HPV-positive patients was mainly enriched in the nucleotide biosynthesis pathway, the signal pathway activity and cell-related protein family expression of cervical cancer samples were significantly upregulated, and the glucose metabolism of vaginal flora in healthy people was more vigorous. The functional remodeling of the flora and the high binding of the SNV genome characteristics mean that as the disease progresses, the vaginal microbes will undergo adaptive changes in the genome and functional levels. Furthermore, the machine learning classification model constructed by fusing SNV features is better than the traditional model constructed by relying only on the abundance of bacterial species. When the prediction model based on SNV was used to distinguish cervical cancer from benign cervical lesions, the AUC value was about 0.86, and the AUC was still stable at 0.79 in the external validation dataset. In summary, microbial SNV characteristics can accurately reflect the strain-level genomic differences and functional heterogeneity that cannot be identified by conventional species classification detection, and can be used as a new and reliable biomarker for the clinical diagnosis and risk stratification of cervical cancer [109].

       ② Specific microbial community composition patterns

Vaginal microbiome composition acts as an important biological marker for diagnosing cervical diseases and classifying clinical risks. A healthy vaginal environment is usually controlled by Lactobacillus species, and *L. crispatus* is the most common dominant strain. On the contrary, people with persistent HPV infection, cervical intraepithelial neoplasia (CIN), or cervical cancer usually have far fewer Lactobacillus, while anaerobic pathogenic bacteria multiply rapidly. Li and Wu’s research proved that the *Gardnerella* content rises sharply when HPV infection occurs and precancerous CIN develops. In addition, a complete meta-analysis completed by Rashwan et al. found that *Porphyromonas*, Campylobacter, Peptococcus, and Anaerococcus all grow in large numbers inside cervical cancer patients. All of these findings show that vaginal microbial profiling can cooperate with traditional clinical indicators such as HPV genotyping, cervical cytology, and pathological testing, so as to build more accurate and detailed risk evaluation models for cervical lesions [100,101]. Combining microbial characteristic features creates a convenient, non-invasive, and highly effective way to conduct personalized disease grading. This new method can help doctors screen high-risk groups at an early stage and carry out targeted intervention to stop serious cervical lesions from getting worse. In essence, diagnosis methods relying on the microbiome optimize traditional detection thinking. Researchers no longer only observe microbial composition simply, but are starting to evaluate the practical influence of pathogenic interactions between microbes and human bodies.

       ③ Microbial Diversity

Microbial diversity is a measurable and trustworthy marker that shows abnormal changes in vaginal microecology. Healthy vaginal flora normally keeps microbial diversity at a low level, and Lactobacillus always takes a strong leading position. On the other hand, HPV infection, long-term viral colonization, CIN lesions, and cervical cancer are strongly linked to higher alpha diversity, which means that the microbial community becomes structurally complicated and is ruled by anaerobic bacteria. Relying only on diversity indices cannot make precise clinical diagnoses. However, if we analyze these indices together with Lactobacillus reduction, the large growth of anaerobic bacteria and inflammatory pathway activation, we can obtain valuable clues about how cervical diseases develop and identify high-risk microecological conditions [79,100,101].

(2) Treatment Response and Recurrence Prediction
  ① Prediction of chemoradiotherapy response

When it comes to how patients respond to treatment, the vaginal microbiome can work as a practical biomarker to judge how well chemoradiotherapy works and to forecast disease recurrence [127]. One study on postmenopausal women with locally advanced cervical cancer found that the overall structure of microbial groups and total bacterial quantity barely changed during concurrent chemoradiotherapy. However, patients who later suffered cancer recurrence had more *Fusobacterium* in their samples taken before treatment. This means that *Fusobacterium* may be connected with treatment resistance and poor prognosis [128]. Vaginal environments controlled by Lactobacillus always help the human body clear HPV naturally more easily and build effective antiviral immune states [127,129]. In comparison, dysbiotic flora filled with large numbers of anaerobes, mainly *Gardnerella vaginalis* and *Atopobium*, usually lead to long-lasting HPV infection, worsening cervical lesions, and weaker treatment effects [128,130,131]. Therefore, adding microbiome profiling to risk classification standards makes predictions more accurate than using traditional clinical markers alone [127]. Some Lactobacillus strains such as *Lactobacillus gasseri* LGV03 can slow down the growth of HPV-positive cervical cancer cells. These strains adjust the body’s immune condition and encourage the production of antiviral interferons (IFN-α and IFN-β), without triggering severe inflammation. This makes them promising materials for immunotherapy [132]. A few studies point out that Vulvovaginal candidiasis (VVC) might lower the chance of catching other HPV subtypes by boosting immunity. Even so, stable overall microecology, especially high levels of Lactobacillus species, greatly cuts down the risk of STIs and helps with better HPV infection recovery [133,134]. These results match earlier research on how *Fusobacterium* suppresses anti-tumor immunity and builds tolerance against chemoradiotherapy, proving that certain microbial groups can help doctors judge chemoradiotherapy effectiveness and predict patient prognosis [135]. In short, stabilizing vaginal microecology—especially raising the quantity of helpful lactobacilli and restraining harmful bacteria including *Lactobacillus iners*—is an important way to improve recovery from HPV infection and obtain better treatment results [136].

       ② Risk of persistent HPV infection and recurrence

Vaginal microecological imbalance, especially microbial communities predominated by *Prevotella*, is tightly correlated with persistent high-risk human papillomavirus (HR-HPV) infection and elevated recurrence risk of cervical cancer [100]. A longitudinal follow-up study of post-treatment cervical cancer patients demonstrated that a *Prevotella*-dominated, highly diverse vaginal microbiota was significantly correlated with persistent HR-HPV detection post-treatment and recurrence within 2–3 years. Among these, *Prevotella*, *Sneathia*, Anaerococcus, Moryella, *Porphyromonas*, Parvimonas, and Negativicoccus were more prominent in patients with recurrence or persistent viral infection, with *Sneathia* amnii identified as a significant biomarker for CIN3 development, and *Porphyromonas* gingivalis shown to colonize cervical squamous cell carcinoma tissues and promote metastasis via JNK/paxillin signaling [137,138,139]. The increased abundance of *Gardnerella vaginalis* also showed a significant association with HPV-related cervical cancer [130]. Another postoperative retrospective study demonstrated that postoperative vaginal pH > 4.5, lactobacillus proportion <50%, and concurrent aerobic vaginitis were all associated with increased recurrence risk. Therefore, dynamic monitoring of key microbial abundance and vaginal ecological indicators post-treatment may help identify patients at high risk of recurrence [110].

#### 4.3.2. Treatment Strategy

Considering the pivotal role of the vaginal microbiome in modulating cervical oncogenesis, HPV persistent infection, and clinical treatment outcomes, targeted microbial intervention strategies have gradually evolved from conceptual exploration to formal clinical validation. These strategies include conventional approaches designed to restore vaginal microbial homeostasis and emerging modalities such as Lactobacillus-based mucosal vaccines, tumor microenvironment-targeted interventions, and metabolic reprogramming. Their primary aims are to promote HPV clearance, facilitate cervical lesion regression, and enhance responses to conventional treatments [27,109,110,111].

(1) Probiotics, prebiotics, and synbiotics:
  ① Restoration of vaginal homeostasis

Oral or intravaginal administration of probiotics, prebiotics, and synbiotics represents the most clinically valuable microecological strategy currently available. This treatment method is widely appealing because it does not require invasive operations and works directly on the local microenvironment, which creates a promising new way to prevent HPV-related diseases and use it as auxiliary treatment. Probiotics including *L. crispatus*, *L. jensenii*, and *L. gasseri* can curb the excessive reproduction of bacteria linked to bacterial vaginosis. They rebuild the dominant position of Lactobacillus, lower vaginal pH to the healthy range of 3.5–4.5, seize nutrients and attachment points from pathogenic bacteria, and generate antibacterial materials such as lactic acid and bacteriocins [101,140]. Relevant research shows that restoring the vaginal flora in this way can speed up HPV clearance and improve cervical cytology results [118]. For patients with cervical precancerous lesions and surgical resection of cervical cancer, postoperative microecological disorders are a key indicator for predicting poor clinical prognosis. A retrospective study of 207 patients with CIN or cervical cancer surgery confirmed that vaginal dysbiosis independently increased the risk of disease recurrence, and screened three high-reliability flora and environmental prognostic indicators: vaginal pH > 4.5, Lactobacillus accounted for less than 50%, and positive aerobic vaginitis test results [141,142]. In addition to predicting postoperative recurrence, more and more studies have shown that the structure of vaginal flora also affects the therapeutic effect of radiotherapy and chemotherapy. A prospective study examined the characteristics of cervical flora in patients with concurrent chemoradiotherapy for cervical cancer [143]. After comparing the recurrent population with the non-recurrent population, it was found that the number of beneficial bacteria in the recurrent patients was significantly reduced, and the stability of the flora structure was destroyed. Among them, *Lactobacillus iners* has outstanding predictive value and has potential as a microbial biomarker for predicting postoperative recurrence [144]. A related study [145] revealed that *Lactobacillus iners*, an obligate L-lactate producer, induced resistance to chemotherapy and radiotherapy in cervical cancer cell lines through metabolic reprogramming, providing mechanistic insight into the clinical association between *L. iners* and poorer survival outcomes in cervical cancer patients. Studies have found that L. iners is likely to mediate chemoradiation resistance, and this conclusion has a high practical clinical reference value. If this conclusion can be verified in a larger prospective cohort study, detection of the vaginal microbiome can be used to guide individualized treatment: for example, screening patients who are suitable for flora regulation intervention before and during concurrent chemoradiotherapy. However, these observational studies cannot rule out the confounding interference caused by indications: patients with L. iners may themselves be superimposed with other risk factors that are not conducive to prognosis. We need to carry out controlled intervention trials, where patients are randomly divided into a flora control group and placebo group, for the synchronous observation of flora changes and clinical efficacy, in order to clarify the causal relationship between the two. In addition, the metabolic mechanism behind the induction of drug resistance by L. iners, especially the role of L-lactate in tumor energy supply and DNA repair, also needs to be explored by rigorous translational medicine research, combining in vitro experimental conclusions with practical clinical efficacy. Current research has confirmed its favorable effects, but more large-scale clinical trials are still required to verify the long-term effectiveness and safety of such microbial interventions among different crowds and various disease phases.

       ② Enhance immune response

Besides rebuilding the vaginal microbiota directly, probiotics can help the human body clear HPV more effectively by adjusting the local immune environment. Its working mechanisms mainly involve adjusting the balance of Th1/Th2 cytokines, optimizing the performance of dendritic cells and antigen-presenting cells, easing long-term inflammatory conditions, and boosting mucosal immune reactions like activating local CD8+ T-cell activity [118]. Lactobacilli create lactic acid and antimicrobial peptides to restrain local inflammation and control cytokine balance. This process strengthens the defense of the mucosal immune barrier and stabilizes immune homeostasis [101]. Healthy microbiota keeps local pro-inflammatory cytokines (IL-1β, IL-12(p40), IL-15, TNF α included) at low levels and keeps the mucosal immune response balanced [146].

All of these mechanisms cooperate closely to raise the body’s general immune ability to eliminate HPV. Consequently, probiotics are recognized to exhibit a synergistic effect with preventive vaccines or therapeutic immunization strategies, providing a theoretical foundation for integrated “microbial ecology–immunity” interventions.

       ③ Adjunctive therapy

Using probiotics together with antibiotics or topical medicines has become a promising way to treat recurring bacterial vaginosis (BV) and long-lasting cervical intraepithelial neoplasia (CIN). Lactin-V is a probiotic product made up of Lactobacillus CTV-05, and it can greatly cut down BV recurrence rates. It protects the vagina by keeping *L. crispatus* steadily settled in the vaginal tract and stopping harmful bacteria from growing back again. More and more studies are focusing on using this probiotic to treat HPV-linked cervical diseases, but there is still not enough direct clinical proof to confirm its curative effect on these illnesses [140]. When probiotics act as auxiliary medicines, they can reduce the damage antibiotics bring to natural helpful microbiota inside the body. They help rebuild a vaginal environment ruled by Lactobacillus, keep the microecology steady, and indirectly protect the defense ability of local mucosal immunity. The treatment effect of this combined therapy mainly depends on well-designed usage plans, such as properly arranging the order of medicine use, the waiting time between treatments and proper dosage, so that antibiotics will not kill the active probiotics. This combined treatment can wipe out pathogenic bacteria and fix vaginal dysbiosis at the same time, yet doctors have not settled on a unified, best clinical usage plan. In conclusion, probiotics work better as supporting treatments for post-screening care, postoperative recovery, and disease recurrence prevention, instead of being used alone to replace mature standard treatments [27,108].

       ④ Issues and Evaluation

Current review papers and clinical trial summaries show that some probiotic preparations work well in rebuilding vaginal homeostasis, cutting down bacterial vaginosis recurrence, and relieving HPV-related lesions. For instance, taking oral *L. crispatus* M247 for 90 days lowers the HPV positive rate by 70% [140]. Most available clinical evidence only comes from preliminary exploratory studies. Probiotics have complicated immunomodulatory effects through many different pathways, so researchers still need further detailed research to understand their exact working mechanisms. Hence, well-designed, placebo-controlled randomized clinical trials are urgently required to verify the long-term efficacy and safety of such combined intervention strategies.

(2) Microbial intervention targeting the tumor microenvironment
  ① Bacterial immunotherapy

More and more researchers are focusing on microbial intervention methods that act on the tumor microenvironment. Besides microbes living on the vaginal epithelial surface, the intratumoral microbiota inside cervical cancer tissue and its immunomodulatory functions have become a popular research topic. The tumor microenvironment of cervical cancer naturally suppresses immune activity; plenty of regulatory T cells (Tregs) and M2-type tumor-associated macrophages (TAMs) gather here. These immune cells help tumors escape immune attacks and speed up cancer development, which shows that microbes living inside tissues play an important role in adjusting tumor immunity [147,148]. Future research may use engineered bacteria to carry immunostimulatory molecules (such as IL-6, IL-8, IL-1β, TNF-α), metabolic regulators, or checkpoint inhibitors as effective ingredients, so as to accurately improve the local immunosuppressive microenvironment. Genetically engineered bacteria, which can find tumor microbial niches and send out immunostimulatory mediators or immune checkpoint inhibitors, form a new bacterial immunotherapy method and are now being tested in preclinical and clinical trials [118]. These engineered strains can release bioactive substances locally, adjust the makeup and functions of tumor-resident microbiota, and change the types and working conditions of immune cells inside tumors. This treatment turns immunosuppressive “cold” tumors into immune-active “hot” tumors, and strengthens the whole body’s anti-tumor immune responses.

In terms of specific mechanisms, metabolites made by Lactobacillus, especially lactate, can turn on the Wnt signaling pathway and raise the core fucosylation levels inside cervical epithelial cells. This molecular change weakens the ability of cervical cancer cells to multiply and spread, revealing a new anti-tumor pathway originating from microbes [149,150,151]. Furthermore, erucic acid, a major metabolite produced by *Lactobacillus crispatus*, can activate the PPAR-δ pathway, boost fatty acid oxidation (FAO), produce large amounts of reactive oxygen species (ROS), and finally trigger ferroptosis in cancer cells to reduce their survival, reproduction, and migration [148]. Even so, this research field is still in the early exploratory stage. Researchers have to solve many problems regarding engineered bacteria, including targeting accuracy, safety, stability inside living bodies, and unwanted side effects. A key task for preclinical research is making sure that engineered bacteria can settle stably in the tumor microenvironment and work steadily without causing whole-body adverse reactions [113,119]. Apart from engineered bacteria, fecal microbiota Transplantation (FMT) is also being studied as a new auxiliary therapy for cervical cancer. Its goal is to adjust microbiota composition, improve immune microenvironment, and control metabolism by introducing healthy “favorable enterotypes” from healthy people [151]. FMT can restrain harmful bacteria, fix intestinal microecology and increase helpful short-chain fatty acids such as butyrate, but it still has obvious difficulties: strict donor selection, unified operation standards, and long-term hidden dangers like pathogen infection and abnormal immune reactions [27,150,151].

       ② Metabolic reprogramming

Microbial metabolites are another very promising target for treatment, and they greatly affect cervicovaginal homeostasis and the development of diseases [152,153,154,155,156]. Research shows that protective metabolites including D-lactic acid can stop cervical stem cells from renewing themselves and slow down disease worsening. In contrast, metabolic networks that trigger inflammation or tumor growth will make long-term viral infection and cancer development more serious [118]. Therefore, the “metabolic reprogramming” treatment method mainly includes adding beneficial metabolites, blocking abnormal metabolic pathways, and adjusting the functions of microbial communities. It changes tumor-cell metabolic pathways by regulating metabolic interactions between hosts and microbes, so as to slow tumor growth and stop tumor metastasis.

The healthy female genital tract is generally colonized predominantly by *Lactobacillus* spp., including *L. crispatus*, *L. gasseri*, *L. jensenii*, and *L. iners*, which sustain microecological homeostasis [153,155,156,157]. These lactobacilli produce plenty of lactic acid by breaking down glycogen, keeping the vaginal pH steady between 3.8 and 4.4. This acidic microenvironment keeps pathogens like Chlamydia trachomatis and *Gardnerella vaginalis* from multiplying too quickly, and it protects the cervical epithelial barrier to stop HPV from invading cells [155,156,158]. Large amounts of D-lactic acid thicken cervicovaginal mucus specifically, trapping viral particles and making it harder for HPV to reach target cells [155].

On the other hand, cervicovaginal dysbiosis features fewer Lactobacillus, higher bacterial diversity, and massive growth of anaerobes such as *Atopobium vaginae*, *Gardnerella vaginalis*, *Prevotella* spp., *Porphyromonas*, and *Fusobacterium*. This unhealthy state has tight links with persistent HPV infection, higher CIN risks, and the development of invasive cervical cancer [155,156,157,158,159,160,161]. This microbial shift changes the local metabolic environment: protective lactic acid drops in quantity, while harmful microbial metabolites including polyamines and bacterial toxins build up gradually [153,155,157]. For example, P. asaccharolytica exhibits metabolic activity related to arachidonic acid (AA), leading to increased levels of 12-HETE, which are associated with MAPK-mediated tumor proliferation [154]. Targeting these metabolic pathways may complement microbiome-based interventions. However, the translation of metabolic reprogramming into clinical practice is complicated by the interconnected nature of microbial metabolic networks and their substantial interindividual variation. Major challenges include identifying metabolites with reproducible tumor-promoting or antitumor effects and developing effective approaches for their targeted delivery or modulation [114].

## 5. Strengths and Limitations

This review synthesized epidemiological, mechanistic, multi-omics, biomarker, and interventional studies spanning HR-HPV infection, cervical intraepithelial neoplasia, and cervical cancer. It also examined host–virus–microbiome interactions, strain-level genetic variation, and microbiome-targeted interventions.

Several limitations should be considered. The narrative review design is susceptible to selection and publication bias. Differences in study populations, sampling protocols, sequencing platforms, bioinformatic pipelines, and outcome definitions limit direct comparison across studies. Most clinical studies are observational or cross-sectional; therefore, the temporal and causal relationships between vaginal dysbiosis, HR-HPV persistence, and cervical lesion progression remain uncertain. Potential confounding factors—including age, hormonal status, menstrual phase, sexual behavior, ethnicity, geographic region, and antibiotic exposure—were not consistently controlled. In addition, many proposed microbial biomarkers and microbiome-targeted interventions have been examined in small, single-center cohorts, with limited external validation and insufficient evidence from adequately powered randomized controlled trials.

## 6. Future Challenges

Here, we collect current data to propose the vaginal microbiome-based strategy for the treatment of HPV infection and cervical cancer. The latest evidence indicates that probiotics in the gut–vagina axis play an important role in clearing HPV [162]. This means that the cross talk between the gut microbiome and vaginal microbiome in HPV infection or cervical cancer should not be neglected in the future. Another challenge is that TCM has been demonstrated to be a promising strategy in the management of clearing HPV infection or as an add-on treatment for cervical cancer. How to make TCM more acceptable based on its precise mechanism and effectiveness is a great issue. Finally, the development and application of artificial intelligent (AI) in decoding the microbiome [163], the prediction of BV-relevant probiotic functional potential in vaginal-derived *Lactobacillus crispatus* strains [164], and exploring the role of the vaginal and fecal microbiome in miscarriage [165] also indicate that AI will be a powerful tool for proceeding to the vaginal microbiome-based strategy for HPV infection or cervical cancer in the future.

## 7. Conclusions

Current evidence associates Lactobacillus depletion, anaerobic bacterial enrichment, increased microbial diversity, and elevated vaginal pH with HR-HPV persistence and more severe cervical disease. Experimental studies suggest that epithelial barrier impairment, altered mucosal immunity, microbial metabolites, and chronic inflammation may contribute to these associations, although direct evidence of causality remains limited. Microbial composition, strain-level variation, and metabolic profiles may have value in cervical lesion detection and risk stratification. However, probiotics, engineered probiotic vaccines, vaginal microbiota transplantation, and other microbiome-targeted interventions remain investigational. Their clinical translation will require standardized sampling and analytical protocols, longitudinal multi-omics studies, external validation of candidate biomarkers, and adequately powered multicenter clinical trials (see Table 2).

The cited literature was grouped into four evidence domains according to study design and the inference that each design can support. Systematic reviews and meta-analyses, largely based on observational studies, report associations between cervicovaginal dysbiosis and HR-HPV infection, persistence, cervical intraepithelial neoplasia, and cervical cancer. Longitudinal studies provide some information on temporal relationships but do not establish causality. Experimental and translational studies support the biological plausibility of epithelial, immune, metabolic, and viral-oncogene-related mechanisms. Microbial, strain-level, methylation, and metabolic signatures remain at the discovery or early validation stage. This evidence map describes the available evidence structure and does not constitute a formal GRADE assessment.

## Figures and Tables

**Figure 1:**
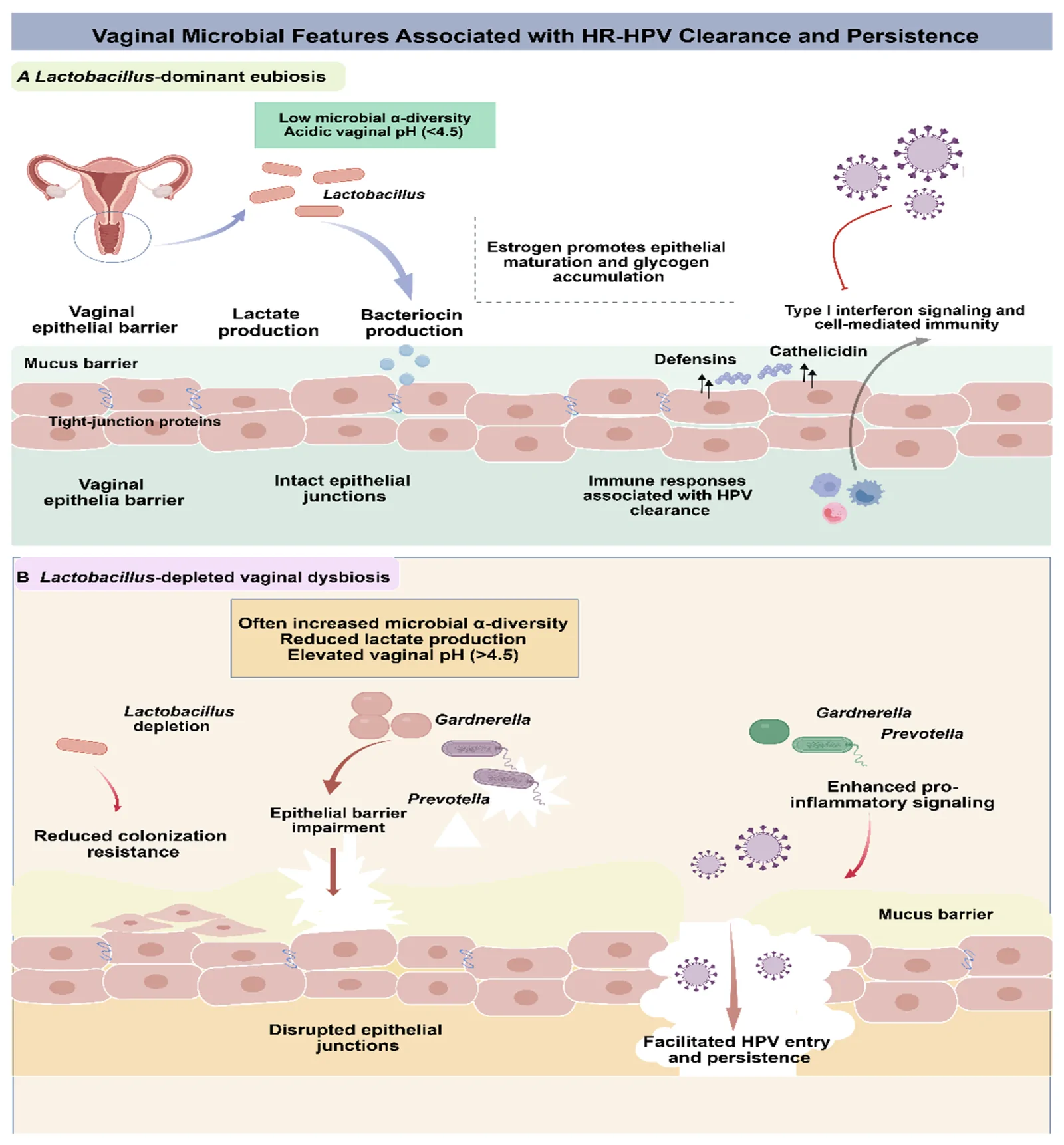
Vaginal Microbial Features Associated with HR-HPV Clearance and Persistence. (**A**) Lactobacillus-dominant communities typically exhibit low α-diversity and an acidic vaginal pH (<4.5). Estrogen supports epithelial maturation and glycogen accumulation, conditions favorable to lactobacilli. Lactate and bacteriocin production contributes to an acidic, colonization-resistant environment. An intact mucus layer and epithelial junctions, together with antimicrobial peptides, type I interferon signaling, and cell-mediated immunity, support mucosal defense and HPV clearance. (**B**) Lactobacillus depletion is often accompanied by reduced lactate production, elevated vaginal pH, greater microbial diversity, and enrichment of anaerobic taxa, including *Gardnerella* and *Prevotella*. Loss of colonization resistance and increased inflammatory signaling may impair the mucus and epithelial barriers, allowing HPV greater access to susceptible basal epithelial cells and favoring viral persistence. These pathways are based on current mechanistic and observational evidence and should not be interpreted as establishing a direct causal relationship between vaginal dysbiosis and persistent HR-HPV infection. Blue arrows denote protective pathways, red arrows denote detrimental pathways, and green/orange shading denotes the Lactobacillus-dominant state and dysbiotic state, respectively.

**Figure 2:**
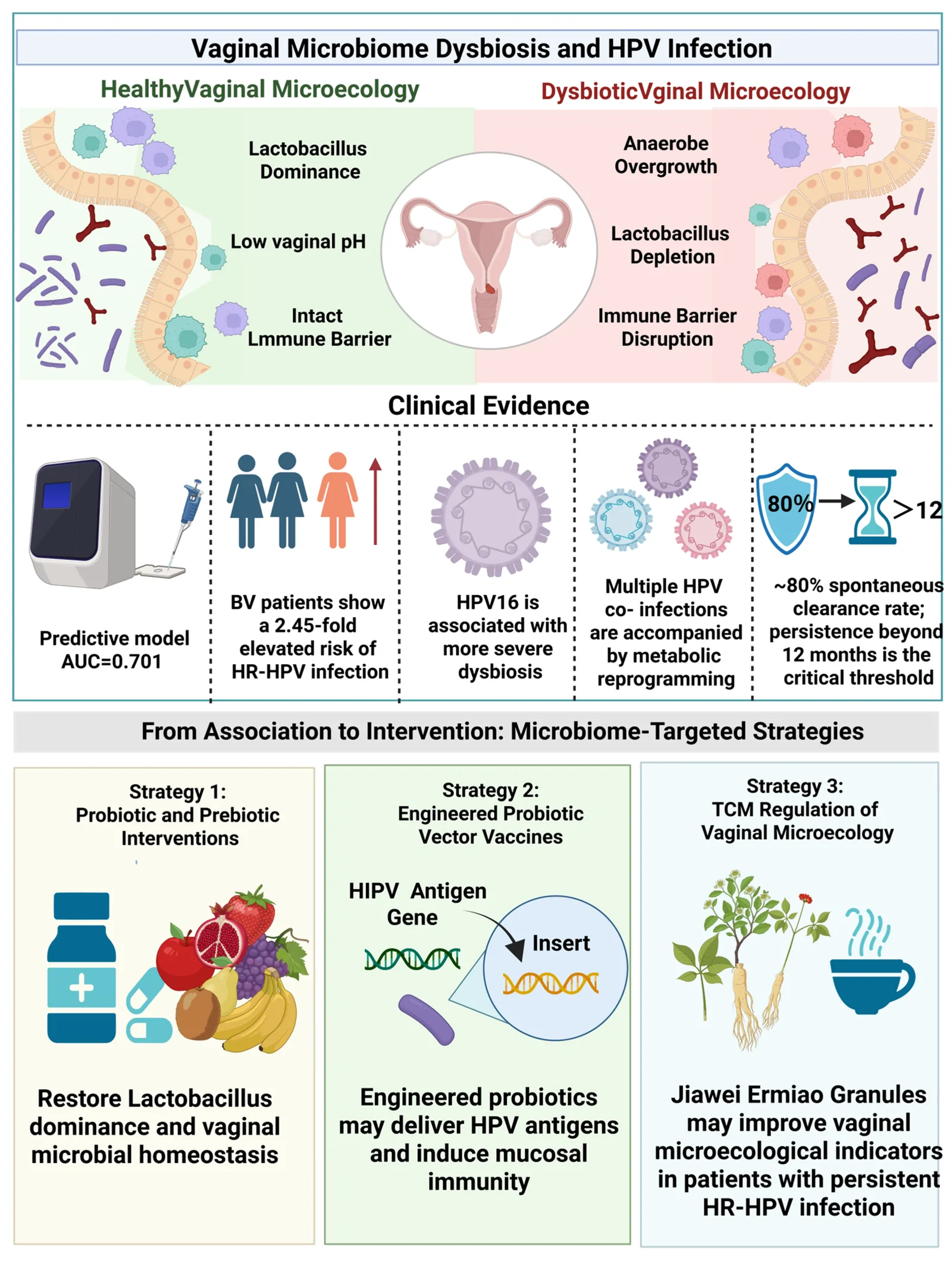
Vaginal microbiome dysbiosis and HPV infection.

**Figure 3:**
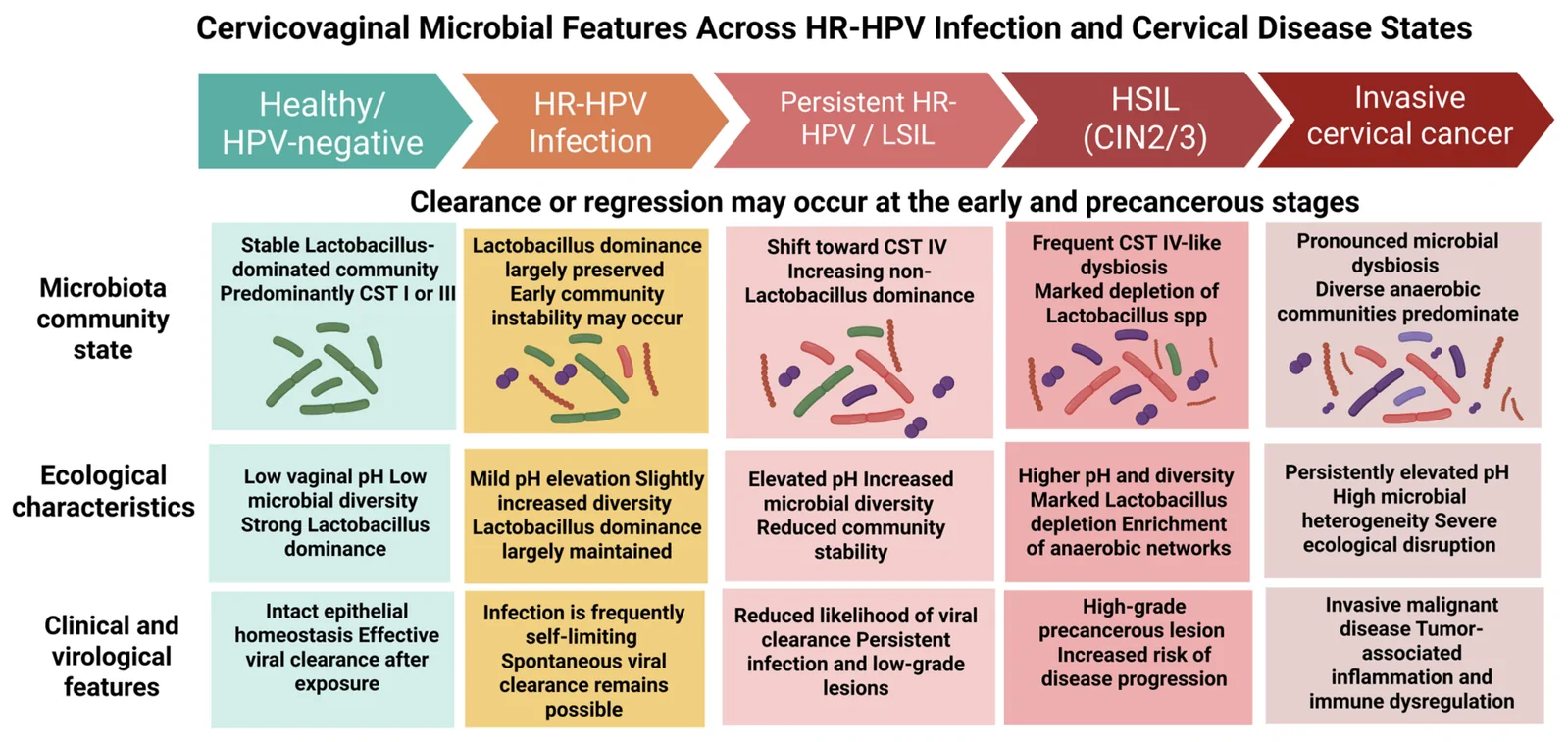
Cervicovaginal microbial features across HR-HPV infection and cervical disease states.

**Figure 4:**
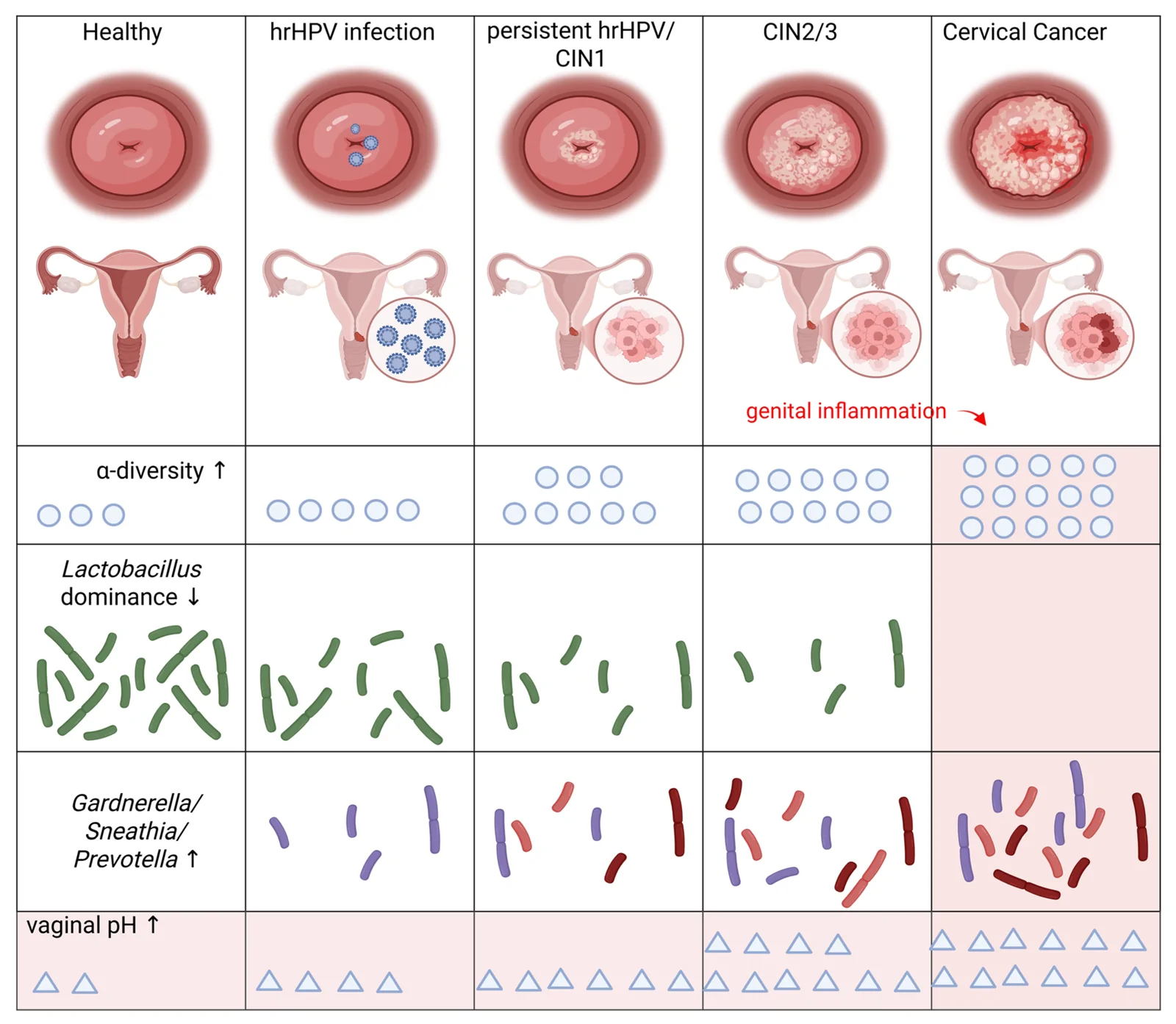
Progressive cervicovaginal microecological dysbiosis across successive clinical stages of HR-HPV infection and cervical carcinogenesis.

**Figure 5:**
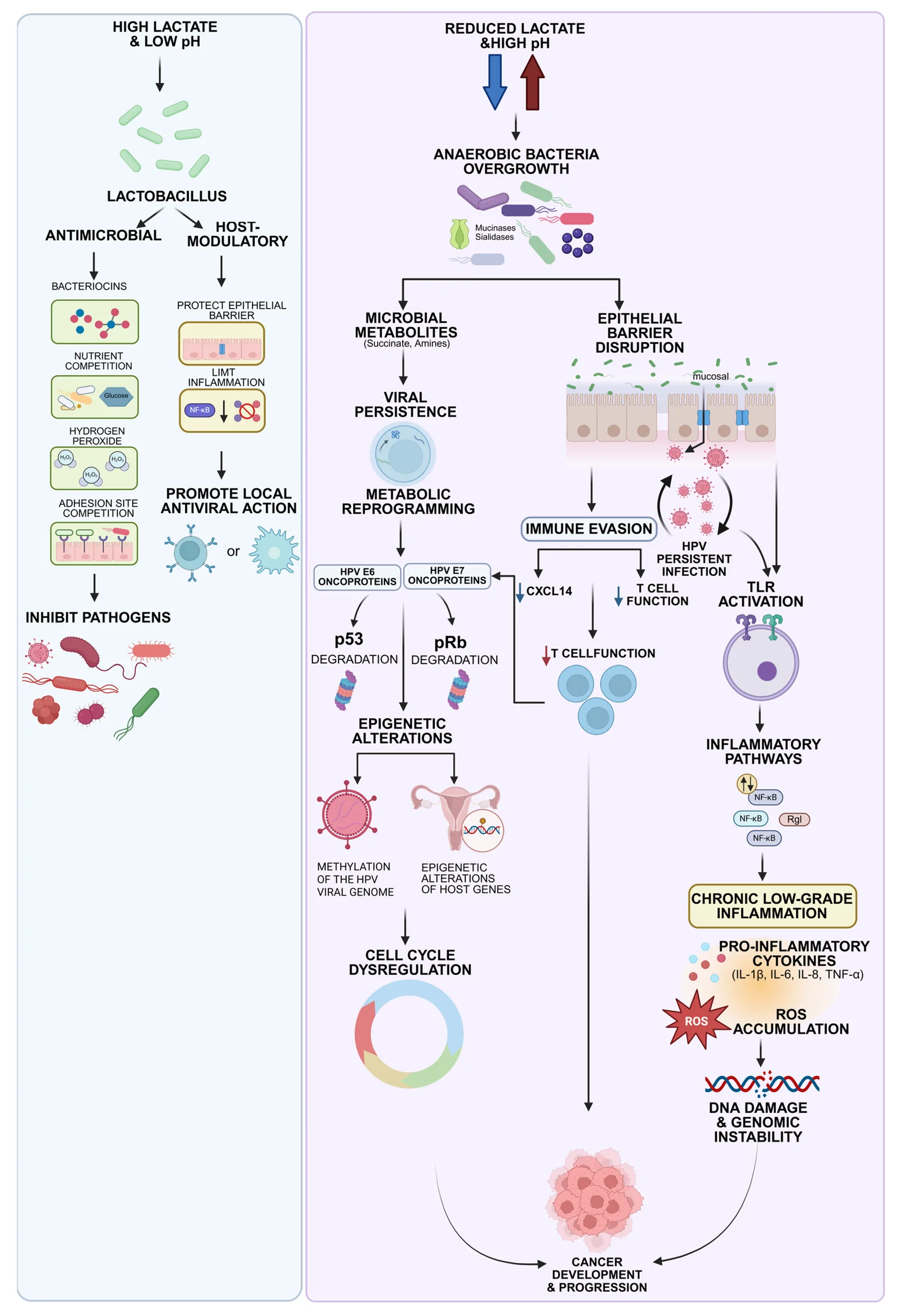
Mechanistic pathways linking vaginal Lactobacillus homeostasis and anaerobic dysbiosis to persistent HPV infection and cervical carcinogenesis. The left panel (light green/blue background) depicts the protective Lactobacillus-dominant pathway, whereas the right panel (light purple background) depicts the dysbiotic pathway. Blue downward and red upward arrows denote decreased lactate and elevated pH, respectively; black solid arrows indicate the direction of causation.

**Table 1:** Evidence base and translational status of microbial and microbiome-modulating interventions for HR-HPV infection and cervical disease.

Intervention	Clinical Evidence Represented	Main Findings	Evidence Limitations and Current Status
Probiotic and Prebiotic Interventions	Small randomized trials and controlled pilot studies [84,87,88]	Changes in HPV-related virological outcomes and vaginal microbial composition have been reported.	Investigational; confirmatory trials required
Engineered Probiotic Vector Vaccines	Phase I, phase I/IIa, and phase IIb clinical studies [90,91,92]	Early studies have evaluated safety, immunogenicity, and lesion-related outcomes.	Early clinical development; therapeutic efficacy not established
Vaginal Microbiota Transplantation (VMT)	Randomized trial for vaginal dysbiosis [6]	Direct evidence for HR-HPV clearance or cervical lesion outcomes is lacking.	Indirect evidence for HPV-related cervical disease
Jiawei Ermiao Granules	Small prospective single-arm study with paired microbiome and metabolomic analyses [96,98]	Within-group changes in HR-HPV status, vaginal microecological indices, microbial composition, and metabolic profiles were reported.	Uncontrolled exploratory evidence; efficacy remains uncertain.

Abbreviations: HR-HPV, high-risk human papillomavirus; VMT, vaginal microbiota transplantation.

**Table 2:** Evidence domains and supported inferences for the cervicovaginal microbiome in HR-HPV persistence and cervical disease.

Evidence Domain	Evidence Base Represented in the Review	Supported Inference	Current Status
Association	Systematic reviews/meta-analyses of observational studies [34,57,59,60,61,81]	Observational studies generally report associations between cervicovaginal dysbiosis and HR-HPV infection, CIN, or cervical cancer.	Generally consistent observational association Causality not established
Temporality	Prospective and longitudinal cohorts [62,71,136,137,142,155]	Longitudinal studies have linked baseline or time-varying microbial profiles to subsequent HPV clearance, persistence, or cervical lesion outcomes.	Limited temporal evidence Causal direction remains uncertain
Mechanisms	Cell, organoid, animal, and translational studies [15,64,89,115,132,145,147,152]	Experimental studies support the biological plausibility of epithelial barrier, immune, metabolic, and viral-oncogene-related pathways.	Experimental and translational support Clinical relevance remains uncertain
Biomarkers	Discovery and retrospective/observational validation studies [69,99,100,109,141,143]	Candidate microbial, SNV, methylation, and metabolic signatures have discriminated disease groups or shown prognostic associations in selected datasets.	Discovery or early validation stage Clinical utility not established

Framework note: This is an evidence map, not a formal GRADE certainty rating. GRADE requires outcome-specific assessment of risk of bias, inconsistency, indirectness, imprecision, and publication bias. Evidence tiers were interpreted by question type using the Oxford CEBM approach; journal impact factor and quartile were not treated as evidence-quality criteria.

## Data Availability

No new data were created or analyzed in this study. Data sharing is not applicable to this article.
